# Supplementary material for: The distribution of technology induced job loss: Evidence from a population-wide study in Norway
Source: PLoS One. 2025 Apr 15;20(4):e0321072. doi: 10.1371/journal.pone.0321072 (PMC11999129; doi:10.1371/journal.pone.0321072)
Supplement: S1 File — (DOCX) [file pone.0321072.s001.docx]

**Supporting information**

**Constructing the routine intensity index (RTI)**

We construct the routine intensity index using the ONET-items suggested by Acemoglu and Autor (2011) (page 1163). We used the 2003 and 2019 versions of ONET, which can be downloaded from <https://www.onetcenter.org/db_releases.html>. Acemoglu and Autor (2011) divide the items into five groups routine manual, routine cognitive, non-routine manual, non-routine cognitive and non-routine interpersonal. To create a score for each occupation along these groups, we average ONET scores, using the importance scores (”IM” in the onet-files). We then translate from SOC2010 to ISCO88 using the bridge provided by the U.S. Bureau of Labor Statistics, <https://www.bls.gov/soc/isco_soc_crosswalk.xls>.

After bridging to ISCO-08, we collapsed to a single dimension RTI-score by standardizing (z-scoring) the following index: *RTI = ln(Routine Manual + Routine Cognitive)− ln(NonRoutine Cognitive + NonRoutineInterpersonal)*
