# Supplementary material for: The distribution of technology induced job loss: Evidence from a population-wide study in Norway
Source: PLoS One. 2025 Apr 15;20(4):e0321072. doi: 10.1371/journal.pone.0321072 (PMC11999129; doi:10.1371/journal.pone.0321072)
Supplement: S1 Table — (DOCX) [file pone.0321072.s002.docx]

**S1 Table. Correlation matrix**

|  | **RTI 2003** | **RTI 2019** | **FOI** |
| --- | --- | --- | --- |
| **RTI 2003** | 1 | 0.85 | 0.68 |
| **RTI 2019** |  | 1 | 0.71 |
| **FOI** |  |  | 1 |

The shows the Pearson correlation between a RTI based on ONET 2003, RTI based on ONET 2019 and the Frey Osbourne Index.
