# Supplementary figures and images for: The distribution of technology induced job loss: Evidence from a population-wide study in Norway
Source: PLoS One. 2025 Apr 15;20(4):e0321072. doi: 10.1371/journal.pone.0321072 (PMC11999129; doi:10.1371/journal.pone.0321072)

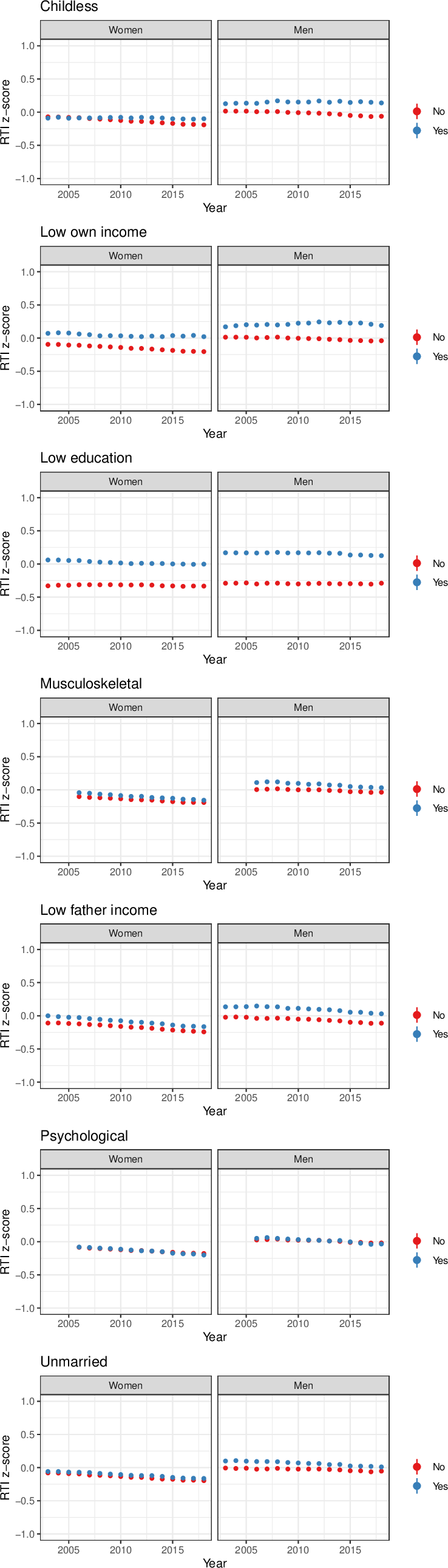

Supplement: S1 Fig — The figure shows the average RTI z-score, stratified by year for 45-year-olds (birth cohorts 1958–1973) by gender. The 95% confidence intervals are indicated by the error bars. (TIF) [file pone.0321072.s003.tif]

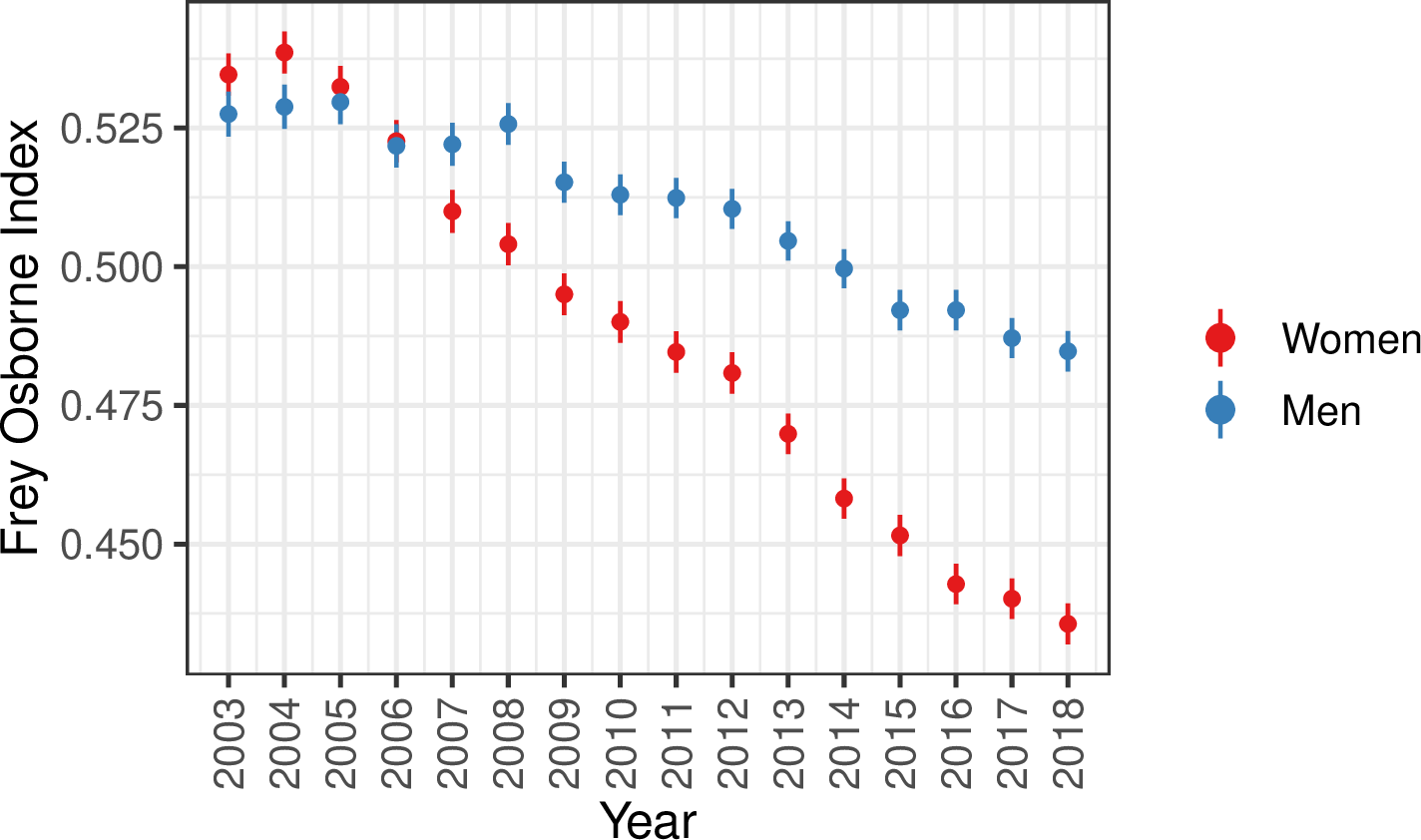

Supplement: S2 Fig — The figure shows the yearly average FOI, for 45-year-olds (birth cohorts 1958–1973) by gender. The 95% confidence intervals are indicated by the error bars. (TIF) [file pone.0321072.s005.tif]

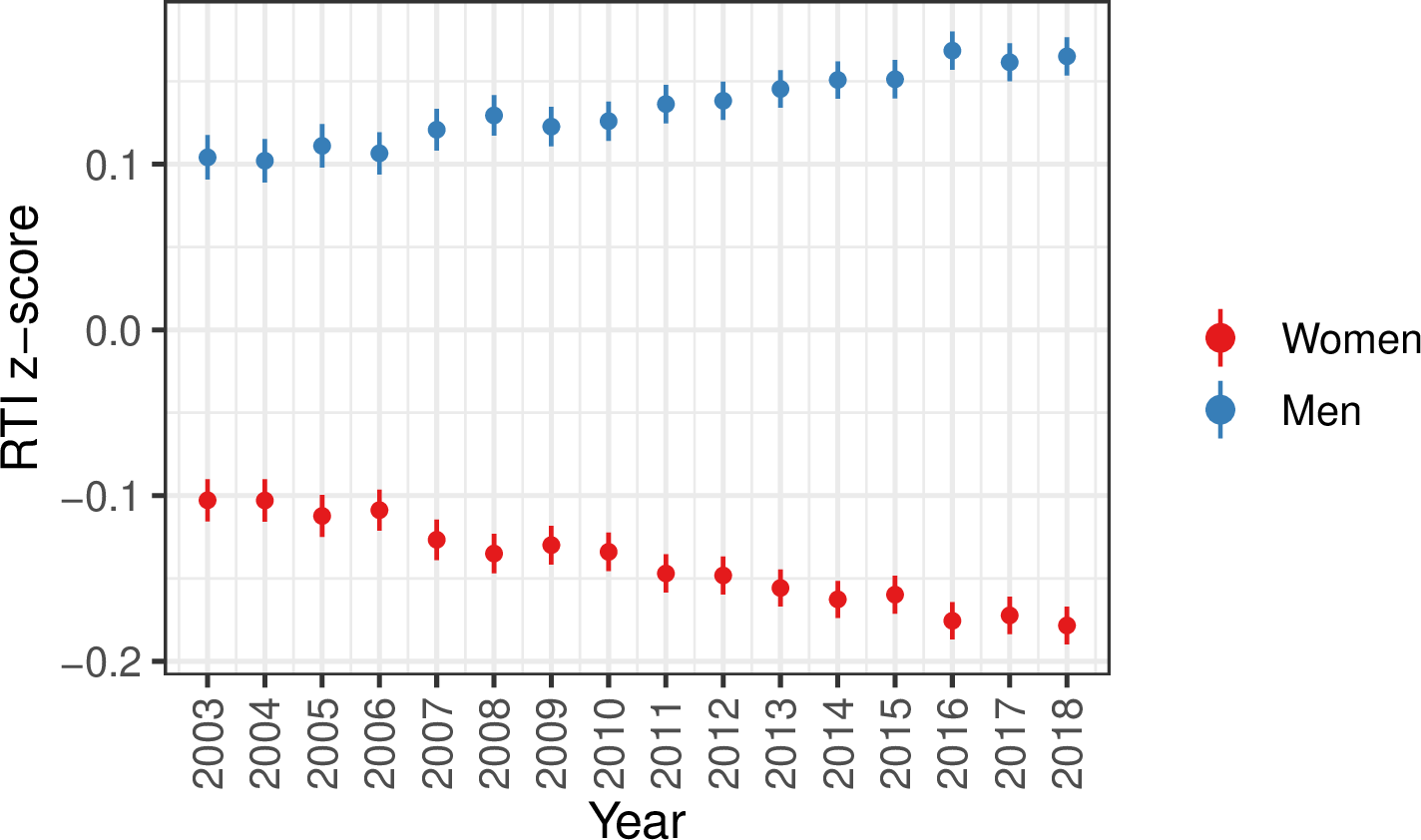

Supplement: S3 Fig — The figure shows the yearly average RTI-2019 z-score, for 45-year-olds (birth cohorts 1958–1973) by gender. The 95% confidence intervals are indicated by the error bars. (TIF) [file pone.0321072.s006.tif]

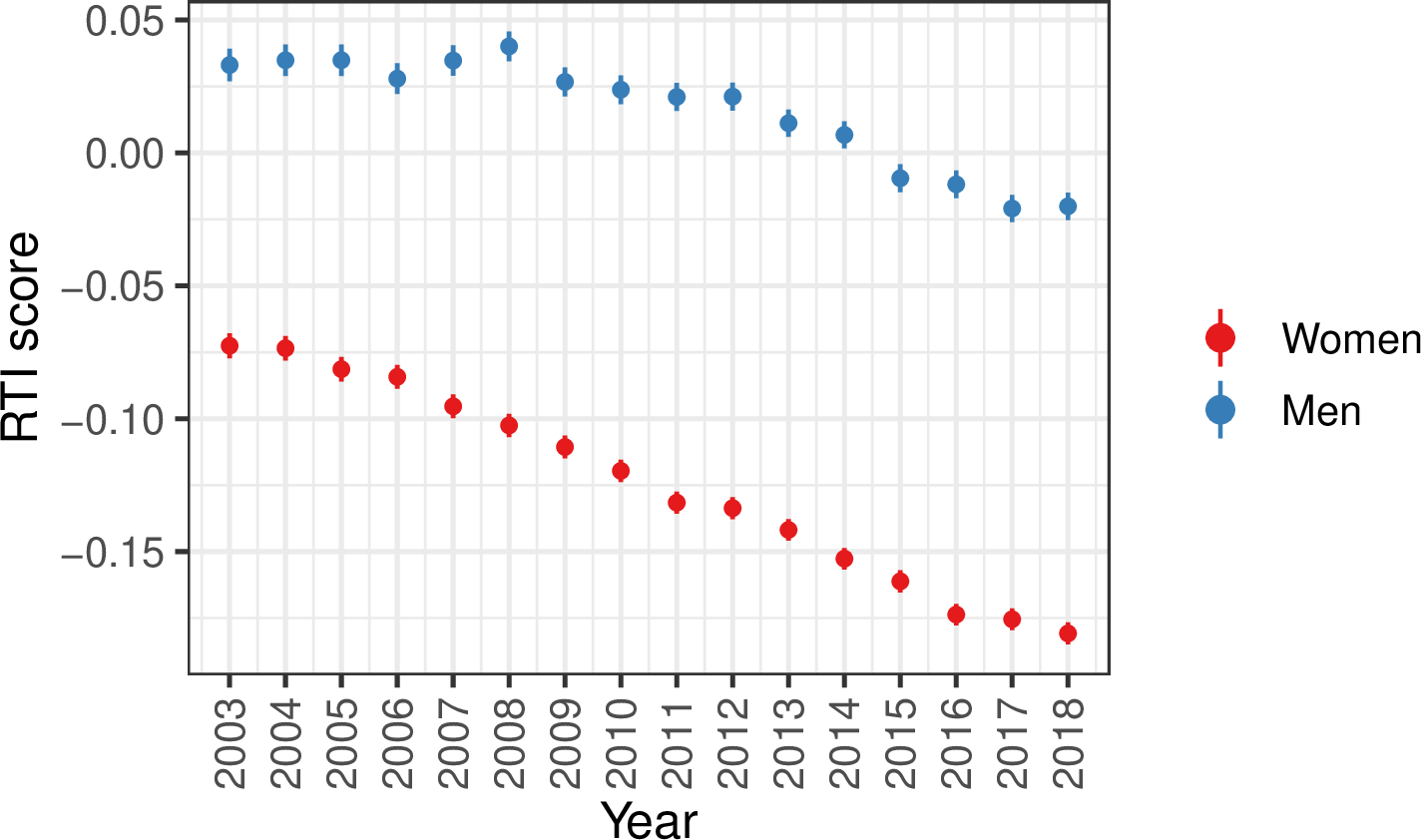

Supplement: S4 Fig — The figure shows the yearly average RTI score, for 45-year-olds (birth cohorts 1958–1973) by gender. The 95% confidence intervals are indicated by the error bars. (TIF) [file pone.0321072.s007.tif]

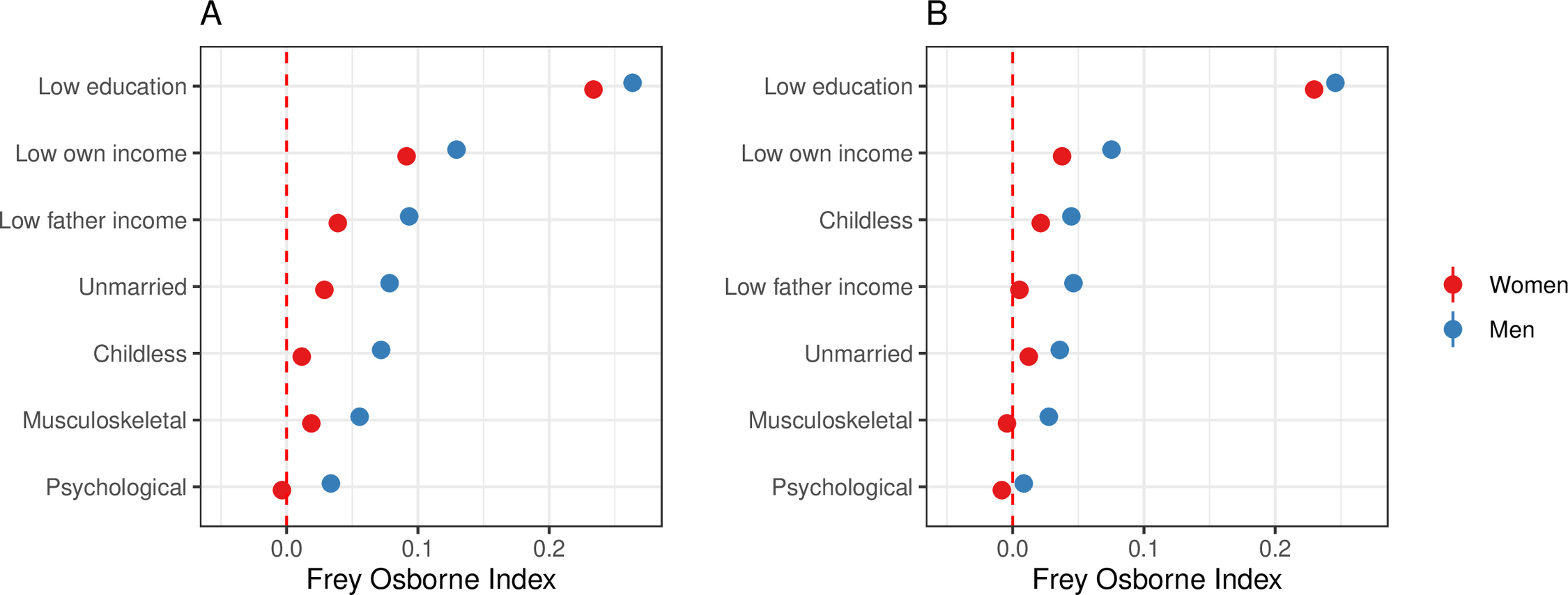

Supplement: S5 Fig — The figure displays regression coefficients from (A) bivariate and (B) multivariate regression models, with the FOI as the outcome variable for all 45-year-olds between 2003 and 2018, separately by gender. The 95% confidence intervals are indicated by the error bars. (TIF) [file pone.0321072.s008.tif]

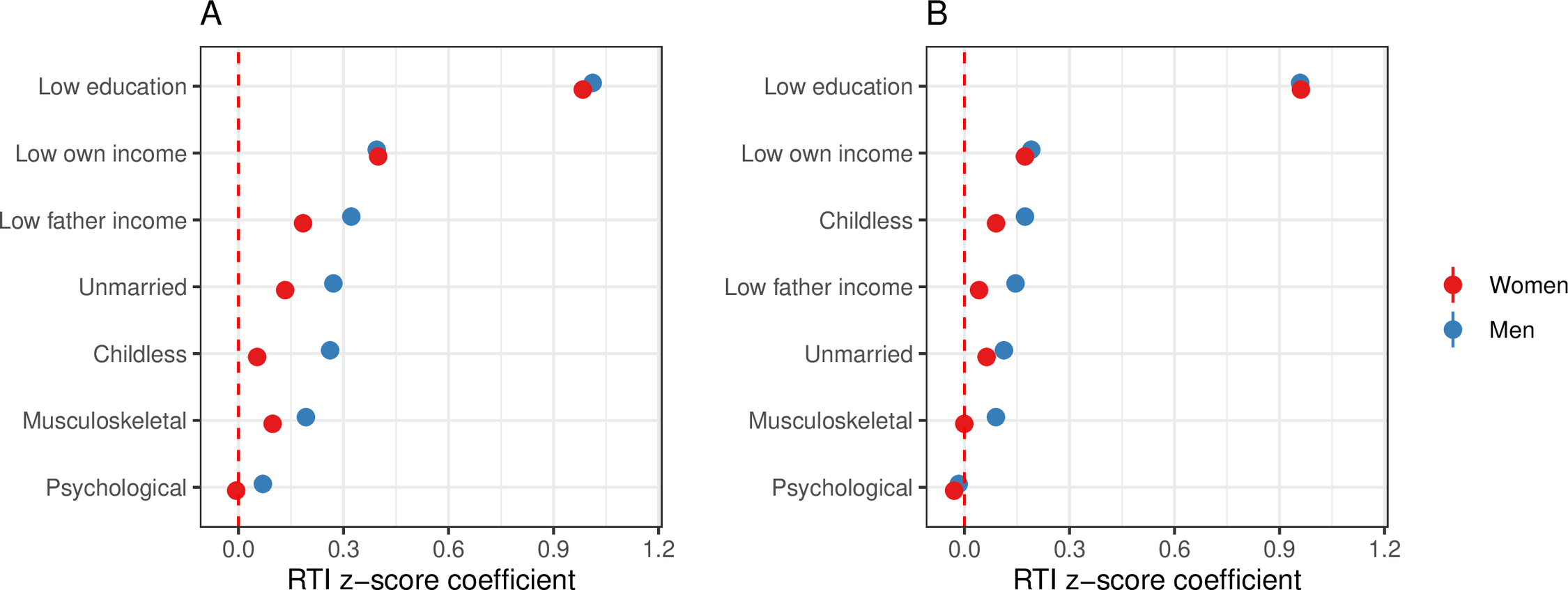

Supplement: S6 Fig — The figure displays regression coefficients from (A) bivariate and (B) multivariate regression models, with the RTI-2019 z-score as the outcome variable for all 45-year-olds between 2003 and 2018, separately by gender. The 95% confidence intervals are indicated by the error bars. (TIF) [file pone.0321072.s009.tif]

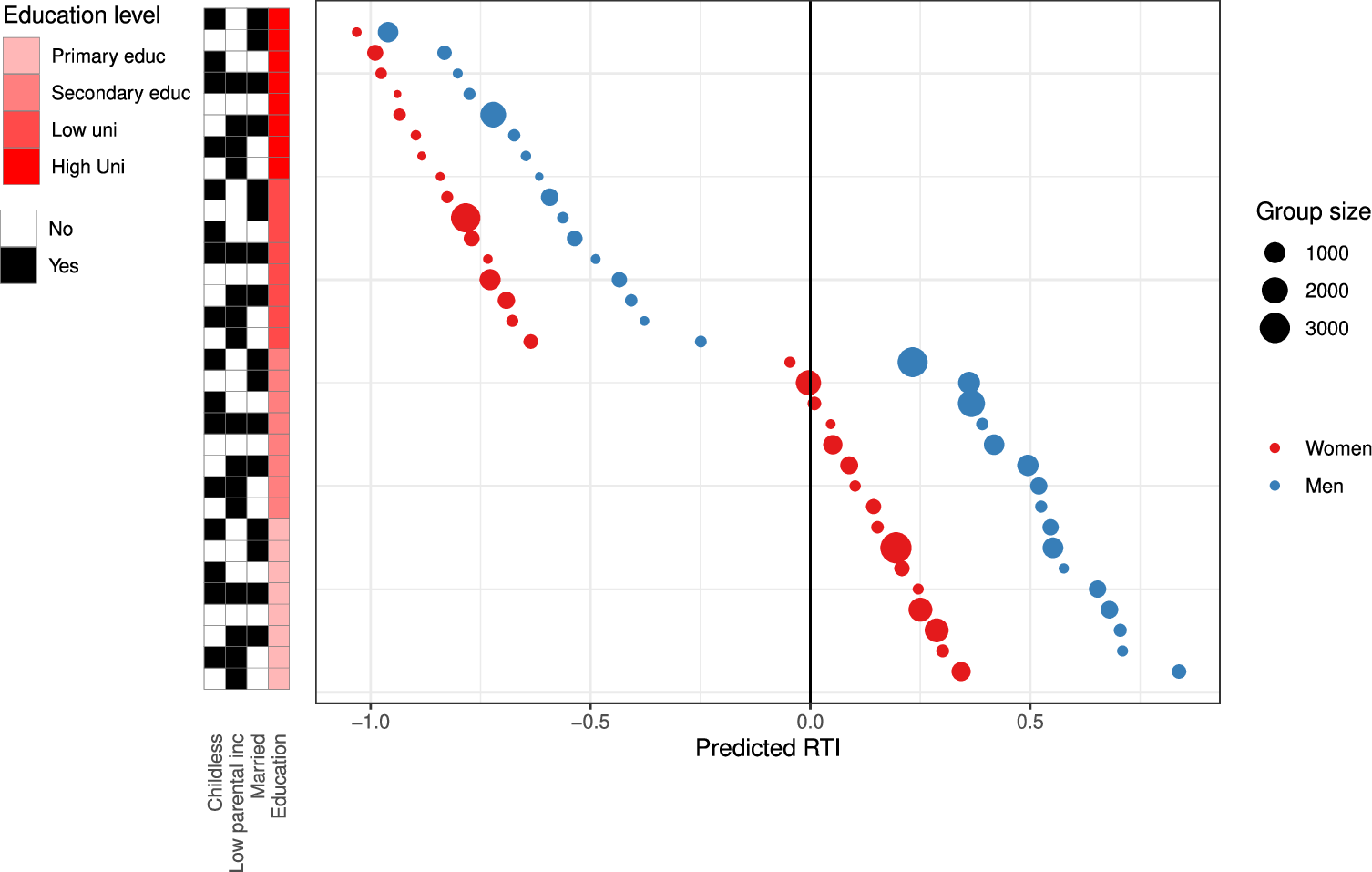

Supplement: S7 Fig — The figure shows the predicted RTI-z-score from a model estimated on the 1958 birth cohort (45 years old in 2003) explaining RTI-z-score with four educational levels, a dummy for childless, a dummy for whether married and a dummy for whether father’s income average income rank between ages 40 and 50 was within the lowest quintile of men of similar age. (TIF) [file pone.0321072.s011.tif]
