# Supplementary material for: The distribution of technology induced job loss: Evidence from a population-wide study in Norway
Source: PLoS One. 2025 Apr 15;20(4):e0321072. doi: 10.1371/journal.pone.0321072 (PMC11999129; doi:10.1371/journal.pone.0321072)
Supplement: S2 Table — (DOCX) [file pone.0321072.s004.docx]

**S2 Table. Gender-specific average RTI-z score among 45-year-olds, 2003-2018.**

| **Year** | **Gender** | **Mean RTI** | **95% CI** | **N** |
| --- | --- | --- | --- | --- |
| **2003** | Women | -0.13 | [-0.14.-0.12] | 24581 |
| **2004** | Women | -0.13 | [-0.14.-0.12] | 24564 |
| **2005** | Women | -0.14 | [-0.15.-0.13] | 24310 |
| **2006** | Women | -0.14 | [-0.15.-0.13] | 25025 |
| **2007** | Women | -0.16 | [-0.17.-0.15] | 25210 |
| **2008** | Women | -0.17 | [-0.18.-0.16] | 26478 |
| **2009** | Women | -0.17 | [-0.18.-0.16] | 27415 |
| **2010** | Women | -0.18 | [-0.19.-0.17] | 27768 |
| **2011** | Women | -0.19 | [-0.2.-0.18] | 28089 |
| **2012** | Women | -0.19 | [-0.2.-0.18] | 28616 |
| **2013** | Women | -0.19 | [-0.2.-0.18] | 29765 |
| **2014** | Women | -0.2 | [-0.21.-0.19] | 30025 |
| **2015** | Women | -0.19 | [-0.2.-0.18] | 28738 |
| **2016** | Women | -0.2 | [-0.21.-0.19] | 29524 |
| **2017** | Women | -0.19 | [-0.2.-0.18] | 29340 |
| **2018** | Women | -0.2 | [-0.21.-0.19] | 28411 |
| **2003** | Men | 0.13 | [0.12.0.14] | 24926 |
| **2004** | Men | 0.13 | [0.12.0.14] | 25251 |
| **2005** | Men | 0.14 | [0.13.0.15] | 24887 |
| **2006** | Men | 0.13 | [0.12.0.14] | 25725 |
| **2007** | Men | 0.15 | [0.14.0.16] | 26506 |
| **2008** | Men | 0.17 | [0.16.0.18] | 27669 |
| **2009** | Men | 0.16 | [0.15.0.17] | 29074 |
| **2010** | Men | 0.17 | [0.16.0.18] | 29530 |
| **2011** | Men | 0.18 | [0.17.0.19] | 30285 |
| **2012** | Men | 0.18 | [0.17.0.19] | 30670 |
| **2013** | Men | 0.18 | [0.17.0.19] | 31902 |
| **2014** | Men | 0.18 | [0.17.0.19] | 32378 |
| **2015** | Men | 0.18 | [0.17.0.19] | 30540 |
| **2016** | Men | 0.19 | [0.18.0.2] | 30965 |
| **2017** | Men | 0.18 | [0.17.0.19] | 31478 |
| **2018** | Men | 0.19 | [0.18.0.2] | 30914 |

The table corresponds to Figure 1.
