# Supplementary material for: The distribution of technology induced job loss: Evidence from a population-wide study in Norway
Source: PLoS One. 2025 Apr 15;20(4):e0321072. doi: 10.1371/journal.pone.0321072 (PMC11999129; doi:10.1371/journal.pone.0321072)
Supplement: S3 Table — (DOCX) [file pone.0321072.s010.docx]

**S3 Table. Results from bivariate and multivariate regression models.**

| **Variable** | **Gender** | **Estimate** | **SE** | **p value** | **Type** |
| --- | --- | --- | --- | --- | --- |
| Childless | Male | 0.25 | 0.005 | 0 | Bivariate |
| Childless | Female | 0.01 | 0.005 | 0.0373 | Bivariate |
| Low education | Male | 1.11 | 0.003 | 0 | Bivariate |
| Low education | Female | 0.82 | 0.002 | 0 | Bivariate |
| Low father income | Male | 0.38 | 0.004 | 0 | Bivariate |
| Low father income | Female | 0.2 | 0.003 | 0 | Bivariate |
| Low own income | Male | 0.36 | 0.007 | 0 | Bivariate |
| Low own income | Female | 0.39 | 0.004 | 0 | Bivariate |
| Musculoskeletal | Male | 0.23 | 0.005 | 0 | Bivariate |
| Musculoskeletal | Female | 0.11 | 0.003 | 0 | Bivariate |
| Psychological | Male | 0.07 | 0.008 | 0 | Bivariate |
| Psychological | Female | 0.03 | 0.004 | 0 | Bivariate |
| Unmarried | Male | 0.3 | 0.004 | 0 | Bivariate |
| Unmarried | Female | 0.12 | 0.003 | 0 | Bivariate |
| Childless | Male | 0.15 | 0.005 | 0 | Multivariate |
| Childless | Female | 0.04 | 0.004 | 0 | Multivariate |
| Low education | Male | 1.05 | 0.003 | 0 | Multivariate |
| Low education | Female | 0.79 | 0.003 | 0 | Multivariate |
| Low father income | Male | 0.19 | 0.004 | 0 | Multivariate |
| Low father income | Female | 0.08 | 0.003 | 0 | Multivariate |
| Low own income | Male | 0.14 | 0.006 | 0 | Multivariate |
| Low own income | Female | 0.2 | 0.004 | 0 | Multivariate |
| Musculoskeletal | Male | 0.12 | 0.004 | 0 | Multivariate |
| Musculoskeletal | Female | 0.03 | 0.003 | 0 | Multivariate |
| Psychological | Male | -0.02 | 0.007 | 5.00E-04 | Multivariate |
| Psychological | Female | 0 | 0.004 | 0.2938 | Multivariate |
| Unmarried | Male | 0.14 | 0.003 | 0 | Multivariate |
| Unmarried | Female | 0.06 | 0.003 | 0 | Multivariate |

Corresponds to Figure 2.
