# Supplementary material for: The distribution of technology induced job loss: Evidence from a population-wide study in Norway
Source: PLoS One. 2025 Apr 15;20(4):e0321072. doi: 10.1371/journal.pone.0321072 (PMC11999129; doi:10.1371/journal.pone.0321072)
Supplement: S4 Table — (DOCX) [file pone.0321072.s012.docx]

**S4 Table. Predicted and average RTI z-scores for men (cf. Fig 4)**

| **Education** | **Marriage** | **Children?** | **Low father inc** | **Average RTI z-score** | **Predicted RTI z-score** | **95% CI** |
| --- | --- | --- | --- | --- | --- | --- |
| Primary education | No | No | Yes | 0.86 | 0.82 | (0.69.0.95) |
| Secondary education | Yes | No | Yes | 0.8 | 0.6 | (0.38.0.82) |
| Secondary education | No | No | Yes | 0.75 | 0.72 | (0.63.0.81) |
| Primary education | No | No | No | 0.75 | 0.67 | (0.57.0.78) |
| Primary education | Yes | No | Yes | 0.71 | 0.7 | (0.34.1.07) |
| Primary education | No | Yes | Yes | 0.7 | 0.67 | (0.58.0.77) |
| Primary education | Yes | Yes | Yes | 0.58 | 0.56 | (0.44.0.67) |
| Primary education | No | Yes | No | 0.55 | 0.53 | (0.45.0.6) |
| Secondary education | No | Yes | Yes | 0.55 | 0.57 | (0.51.0.64) |
| Secondary education | Yes | Yes | Yes | 0.53 | 0.46 | (0.4.0.52) |
| Secondary education | No | No | No | 0.52 | 0.57 | (0.51.0.64) |
| Secondary education | No | Yes | No | 0.45 | 0.43 | (0.39.0.47) |
| Secondary education | Yes | No | No | 0.44 | 0.46 | (0.3.0.62) |
| Primary education | Yes | Yes | No | 0.32 | 0.41 | (0.33.0.49) |
| Primary education | Yes | No | No | 0.3 | 0.56 | (0.29.0.83) |
| Secondary education | Yes | Yes | No | 0.28 | 0.31 | (0.28.0.35) |
| Low university | No | No | Yes | -0.22 | -0.17 | (-0.3.-0.04) |
| Low university | No | No | No | -0.28 | -0.32 | (-0.38.-0.25) |
| Low university | Yes | No | No | -0.32 | -0.43 | (-0.57.-0.29) |
| High university | No | No | Yes | -0.4 | -0.34 | (-0.55.-0.13) |
| Low university | No | Yes | Yes | -0.41 | -0.32 | (-0.4.-0.23) |
| Low university | No | Yes | No | -0.46 | -0.46 | (-0.5.-0.42) |
| Low university | Yes | No | Yes | -0.52 | -0.29 | (-0.6.0.03) |
| High university | No | No | No | -0.52 | -0.49 | (-0.56.-0.41) |
| Low university | Yes | Yes | Yes | -0.53 | -0.43 | (-0.49.-0.37) |
| High university | No | Yes | Yes | -0.55 | -0.49 | (-0.63.-0.35) |
| Low university | Yes | Yes | No | -0.56 | -0.58 | (-0.61.-0.55) |
| High university | Yes | Yes | Yes | -0.69 | -0.6 | (-0.69.-0.52) |
| High university | No | Yes | No | -0.69 | -0.63 | (-0.68.-0.59) |
| High university | Yes | Yes | No | -0.7 | -0.75 | (-0.78.-0.72) |
| High university | Yes | No | Yes | -0.72 | -0.46 | (-1.01.0.09) |
| High university | Yes | No | No | -0.73 | -0.6 | (-0.76.-0.45) |
