# Supplementary material for: The distribution of technology induced job loss: Evidence from a population-wide study in Norway
Source: PLoS One. 2025 Apr 15;20(4):e0321072. doi: 10.1371/journal.pone.0321072 (PMC11999129; doi:10.1371/journal.pone.0321072)
Supplement: S5 Table — (DOCX) [file pone.0321072.s013.docx]

**S5 Table. Predicted and average RTI z-scores for women (cf. Fig 4)**

| **Education** | **Marriage** | **Children?** | **Low father inc** | **Average RTI z-score** | **Predicted RTI z-score** | **95% CI** |
| --- | --- | --- | --- | --- | --- | --- |
| Primary education | No | No | Yes | 0.41 | 0.37 | (0.17.0.58) |
| Primary education | No | Yes | Yes | 0.32 | 0.27 | (0.19.0.35) |
| Primary education | No | No | No | 0.3 | 0.31 | (0.14.0.48) |
| Primary education | Yes | No | No | 0.29 | 0.25 | (-0.12.0.62) |
| Secondary education | Yes | No | Yes | 0.26 | 0.24 | (0.02.0.46) |
| Primary education | Yes | Yes | Yes | 0.24 | 0.21 | (0.12.0.3) |
| Primary education | No | Yes | No | 0.22 | 0.2 | (0.15.0.26) |
| Secondary education | No | Yes | Yes | 0.2 | 0.19 | (0.14.0.24) |
| Secondary education | No | No | Yes | 0.2 | 0.3 | (0.16.0.43) |
| Secondary education | No | No | No | 0.18 | 0.23 | (0.14.0.32) |
| Secondary education | Yes | Yes | Yes | 0.17 | 0.13 | (0.08.0.18) |
| Secondary education | Yes | No | No | 0.13 | 0.17 | (0.0.34) |
| Secondary education | No | Yes | No | 0.12 | 0.13 | (0.09.0.16) |
| Primary education | Yes | Yes | No | 0.08 | 0.14 | (0.08.0.21) |
| Secondary education | Yes | Yes | No | 0.07 | 0.07 | (0.04.0.1) |
| Primary education | Yes | No | Yes | 0.05 | 0.32 | (-0.15.0.78) |
| Low university | No | No | Yes | -0.34 | -0.4 | (-0.51.-0.29) |
| Low university | No | No | No | -0.4 | -0.47 | (-0.53.-0.41) |
| Low university | Yes | No | No | -0.44 | -0.52 | (-0.65.-0.4) |
| High university | No | No | Yes | -0.48 | -0.57 | (-0.72.-0.41) |
| Low university | No | Yes | Yes | -0.52 | -0.51 | (-0.55.-0.46) |
| High university | Yes | No | Yes | -0.55 | -0.62 | (-0.98.-0.26) |
| Low university | Yes | No | Yes | -0.55 | -0.46 | (-0.66.-0.25) |
| Low university | No | Yes | No | -0.57 | -0.57 | (-0.6.-0.55) |
| Low university | Yes | Yes | Yes | -0.6 | -0.56 | (-0.6.-0.53) |
| Low university | Yes | Yes | No | -0.63 | -0.63 | (-0.65.-0.61) |
| High university | Yes | No | No | -0.71 | -0.69 | (-0.85.-0.53) |
| High university | No | Yes | No | -0.72 | -0.74 | (-0.78.-0.7) |
| High university | No | No | No | -0.74 | -0.63 | (-0.73.-0.54) |
| High university | Yes | Yes | Yes | -0.77 | -0.73 | (-0.8.-0.65) |
| High university | No | Yes | Yes | -0.78 | -0.67 | (-0.76.-0.58) |
| High university | Yes | Yes | No | -0.78 | -0.8 | (-0.83.-0.77) |
