# Supplementary material for: The distribution of technology induced job loss: Evidence from a population-wide study in Norway
Source: PLoS One. 2025 Apr 15;20(4):e0321072. doi: 10.1371/journal.pone.0321072 (PMC11999129; doi:10.1371/journal.pone.0321072)
Supplement: Attachment — Submitted filename: Response to reviewers.docx [file pone.0321072.s016.docx]

**PONE-D-23-25076**

**Response letter**

We want to thank the reviewers for taking the time to comment on our study and provide thoughtful comments and constructive feedback. Your insights have been valuable in improving the quality of our study.

In the following we respond to each of your comments. In situations where we cite our revised manuscript and have added sentences to previously existing text, we have highlighted changes/additions in yellow.

Note: the line numbers indicating where changes were made correspond with the tracked changes version of the manuscript (not the line numbers in the revised without tracked changes).

**Reviewer #1:**

1) *Limited Generalizability: The study focuses solely on Norway, which might limit its applicability to other socio-economic contexts.*

**Response:** The generalizability of the study is now clarified in two ways: (1) a statement regarding potential limited generalizability is mentioned in the discussion section of the paper, (2) results from other countries brought into the discussion section.

Specifically, the discussion section now contains a “strengths and weaknesses” subsection that notes the following with regards to generalizability (line 309):

“[...] At the same time, our study has several weaknesses. First, the study has limited generalizability due to its focus on Norway, a country with unique characteristics related to gender roles, institutional environments, and industry structure.”

The discussion section is further expanded with the following (line 284):

“Gender disparities in the risk of automation have also been found in other studies covering several European countries [21]. Interestingly, whether men or women are found to be at the higher risk of automation varies across countries, with the Nordic countries having a higher risk among men. There could be several reasons for such differences across countries, for example related to the Nordic countries having strong gender equality norms, high growth in the education level among women, and lower levels of female part-time work. Moreover, our study covers prime-aged individuals, and the risk likely varies substantially across age. The importance of age is documented in a recent study assessing the causal impact of robot- and information technology across several European countries. While the impact of these technologies on employment were more negative among older women, compared to older men, the impact in prime age (age 30-49) was considerably more negative for men than for women [22].”

2) *Data Sensitivity: The paper uses sensitive data that cannot be shared, which might limit the reproducibility of the study.*

**Response:** The study is not based on proprietary data, but uses sensitive administrative data that the Norwegian Government makes available for researchers under specific conditions. It consists of detailed individual-level information across a wide range of domains (healthcare utilization, income, education, family etc.). While we are prohibited from sharing the data directly, other researchers can gain access under similar terms by applying to “Helsedata.no” at the Norwegian Institute of Public Health and Statistics Norway, and fulfilling the required conditions.

We have rewritten the “Availability of data and materials” to clarify this aspect (line 332):

“The data used in this study encompasses educational outcomes, income, employment records, health records and demographic information for entire cohorts of the Norwegian population. These data were made available on loan for research purposes. Other researchers may apply for access to the same sources - see “[helsedata.no/en](http://helsedata.no/en)” and https://www.ssb.no/en/data-til-forskning/utlan-av-data-til-forskere.”

3) *Complexity: The paper seems to be quite dense and might benefit from a simplified explanation of the key findings for a broader audience.*

**Response:** We have simplified our summary of key findings to better fit a broader audience in the first paragraph of the discussion and in the conclusion.

The first paragraph of the discussion has been rewritten to (line 251):

“Our study has four main findings. First, the risk of automation was higher among men than women. This difference increased from 2003 to 2018. Second, the risk of automation was higher among individuals with low socioeconomic status and less social support in the form of a partner and children. Third, the main reason for the increasing gender gap was the growing gender differences in educational attainment. In summary, our study suggests that automation may exacerbate existing social inequalities and create a more unstable job market situation for men that may already struggle financially and socially.”

4) *Lack of Qualitative Insights: While the paper is strong in its quantitative analysis, incorporating qualitative data could provide a more holistic view.*

**Response:** Our study is purely quantitative study. We added the following to the limitations subsection (line 311):

“[...] Second, the scope of the study is limited to what can be quantitatively measured in administrative registers and may not capture important experiential aspects related to technological development that could be measured with qualitative methods.”

5) *Future Projections: The paper could be strengthened by including future projections based on current trends, which would be valuable for policymakers.*

**Response:** We see this as outside the scope of the current paper. Given the rapidly moving field of artificial intelligence - e.g., the recent introduction of large language models, such as ChatGPT and multimodal models - the scope and potential of automation is likely to have shifted qualitatively. Against this backdrop, we think there is limited relevance of projecting. We now clarify this stance in the discussion (last part of the first paragraph, line 255):

“In summary, our study suggests that automation may exacerbate existing social inequalities and create a more unstable job market situation for men that may already struggle financially and socially. And, if disparities in economic and social opportunity are allowed to grow without mitigating policies, they have the potential to create social unrest and political polarization. At the same time, the nature of technological development changes rapidly, as evidenced by the introduction of large language models and multimodal models in recent years. These new developments may have further increased the scope of automation, potentially increasing the scope of routine tasks to also cover language-based tasks such as report writing and analysis.”

6) *Ethical Considerations: Given that the paper deals with sensitive data and has potential policy implications, a section discussing the ethical considerations would be beneficial.*

**Response:** We have added a separate section that discusses ethical considerations (lines 150-158).

7) *Comparative Analysis: It might be useful to compare the situation in Norway with other countries to provide a more comprehensive view.*

**Response:** We now place our results in the context of related studies from other countries in the discussion section. As noted above in point 1, we also added a statement about the limitations to generalizability in the limitations section.

**Reviewer #2:**

1) *Methodological Clarification: While the paper uses the RTI index and mentions the Frey-Osborne index, it would benefit from a more detailed explanation of why the RTI was chosen over other indices and the specific advantages it offers.*

**Response:** We have expanded the section on the RTI measure to clarify why we chose RTI, but it should be noted that the main results are robust to the choice of RTI or the Frey-Osborne index. This is noted in lines 92-95: “S1 Table shows that all these measures of structural change are highly correlated, and our main conclusions are therefore independent of the selected measure.“

The highlighted sentences below show the additions made (line 80):

“The RTI is one of two commonly used measures used to assess the risk of automation, with the other being the Frey-Osborne index (FOI) [13, 14]. The main difference between these measures is that the RTI is task-based – it assesses to what extent a job consists of tasks that could be automated – while the FOI is based on expert assessment of occupations. The measures are correlated, but RTI has a stronger theoretical foundation, allows for occupational scores to change with their task content [15], has done comparatively better in predictive tests [16], and has been used in an extensive literature on skill biased technological change [2, 13, 17-19] (see [20] and [21] for a description and discussion of the difference between these measures, and other alternative measures used in the literature).

2) *Comparative Analysis: It might be beneficial to compare the situation in Norway with other countries to provide a broader context and understand if the findings are unique to Norway or part of a global trend.*

**Response:** We now discuss the Norwegian results in the context of studies from other countries in the discussion section, while also noting some limitations to generalizability across national contexts in the strengths and weaknesses subsection of the discussion. More specifically:

The context from other studies in the discussion section (line 284):

“Gender disparities in the risk of automation have also been found in other studies covering several European countries [21]. Interestingly, whether men or women are found to be at the higher risk of automation varies across countries, with the Nordic countries having a higher risk among men. There could be several reasons for such differences across countries, for example related to the Nordic countries having strong gender equality norms, high growth in the education level among women, and lower levels of female part-time work. Moreover, our study covers prime-aged individuals, and the risk likely varies substantially across age. The importance of age is documented in a recent study assessing the causal impact of robot- and information technology across several European countries. While the impact of these technologies on employment were more negative among older women, compared to older men, the impact in prime age (age 30-49) was considerably more negative for men than for women [22].”

From the strengths and weaknesses subsection (line 309):

“[...] At the same time, our study has several weaknesses. First, the study has limited generalizability due to its focus on Norway, a country with unique characteristics related to gender roles, institutional environments, and industry structure.”

3) *Potential Bias: The paper could address potential biases in the data, especially given that the data is from administrative registers. Are there any groups that might be underrepresented?*

**Response:** The administrative registers are similar to those used for tax purposes etc., which means they cover all employees in the country. The only workers excluded will be those who are either self-employed or working in the non-formal illegal economy. We now clarify this in the new strength and weaknesses subsection of the discussion (line 305):

“The main strength of our study is that it covers the full population of employed 45-year-old individuals in Norway over a 15-year period across several important characteristics related to health, financial position, education, and family formation. Hence, in contrast to most other studies on this topic which uses survey data, our study does not suffer from selection bias. At the same time, our study has several weaknesses…”

4) *Future Implications: While the paper touches upon the potential negative impacts, a more in-depth exploration of the long-term implications of these findings would be valuable. For instance, what might be the societal consequences if these trends continue?*

**Response:** While interesting and important, we see future implications as beyond the scope of the present study. Recent developments in generative artificial intelligence (large language models such as ChatGPT and multimodal models) have caused a qualitative shift in the nature of automatable tasks, and are likely to substantively change the relative automation risks of different occupations in the future. Projections would also need to consider plausible social and governmental responses and how these may affect outcomes and developmental paths.

We have added the following to first paragraph of the discussion (line 255):

“In summary, our study suggests that automation may exacerbate existing social inequalities and create a more unstable job market situation for men that may already struggle financially and socially. And, if disparities in economic and social opportunity are allowed to grow without mitigating policies, they have the potential to create social unrest and political polarization. At the same time, the nature of technological development changes rapidly, as evidenced by the introduction of large language models and multimodal models in recent years. These new developments may have further increased the scope of automation, potentially increasing the scope of routine tasks to also cover language-based tasks such as report writing and analysis.”

5) *Recommendations: The conclusion section could benefit from specific policy recommendations based on the findings. For instance, what kind of educational or training programs might help mitigate the risks identified?*

**Response:** Our study design and the scope of our paper does not allow us to identify policies that would causally improve outcomes relative to some “business as usual” trajectory. Therefore, a clear recommendation would be overreaching. We have therefore added a statement about the importance of continuing to monitor developments and evaluate programs aimed at supporting individuals that are particularly negatively affected by technological advancements (line 319):

“Given the increasing disparities identified in this study, policy makers should monitor the development in job market opportunities for various subgroups of the working-age population, and evaluate programs aimed at supporting individuals that are particularly negatively affected.”
