# Supplementary material for: The distribution of technology induced job loss: Evidence from a population-wide study in Norway
Source: PLoS One. 2025 Apr 15;20(4):e0321072. doi: 10.1371/journal.pone.0321072 (PMC11999129; doi:10.1371/journal.pone.0321072)
Supplement: Attachment — Submitted filename: Comments to authors.docx [file pone.0321072.s015.docx]

The manuscript titled “**The Distribution of Technology-Induced Job Loss: Evidence from a Population-wide Study in Norway**” explores a significant and timely topic—the impact of automation on job loss and socioeconomic disparities, with a particular focus on gender inequality. The study uses a large and comprehensive dataset from Norway, providing robust evidence to support its conclusions. Overall, the manuscript is well-written, but some revisions are required to improve clarity, expand the discussion, and address methodological details.

**Strengths:**

Using a comprehensive individual-level dataset allows for detailed analysis and lends credibility to the findings. The longitudinal nature of the data (2003-2018) enhances the robustness of the conclusions. The paper successfully highlights growing gender disparities in occupational routine intensity, particularly among individuals with low socioeconomic status, making a valuable contribution to labor economics and automation literature. The attribution analysis in explaining the increasing gender gap is a strong aspect of the paper, offering insight into the reasons behind these disparities.

**Abstract Structure**: The abstract should clearly outline the research objective, methodology, key findings, and implications. Consider adding a brief sentence at the end regarding policy implications or next steps to round it off.

1. Introduction: Ensure the introduction highlights the research gap more explicitly. This will better establish the context for the study and its relevance to the field. Furthermore, summarizing the hypothesis or key research questions can sharpen the focus.

**Methodology Clarification:**

1. Clearly specify the reasoning behind using the RTI index over other measures of automation risk like the Frey-Osborne Index, even though both are discussed. Include a brief comparison in the methodology section, not just in the results section, to improve clarity for readers.
2. Add more detail about how data access and linkage were done (e.g., anonymization procedures and encryption).

**Data Presentation:**

1. Ensure consistency in reporting the variables. The terms used for categories (e.g., education, income) should remain consistent throughout.
2. Consider providing more detailed tables or visual aids in the results section for critical variables to enhance clarity.

**Discussion Section:**

1. Address the limitations more extensively, particularly focusing on the external validity of the findings outside of Norway.
2. Expand on the implications of automation beyond just Norway to make the discussion more globally relevant. Including comparisons to other European countries or worldwide trends would enhance the broader significance of the findings.

**Figures and Tables:**

1. Ensure all figures and tables are fully labelled and include clear legends. Some figures (like those comparing gender over time) could benefit from a more detailed description in the figure caption to ensure they are standalone and clear to readers.
2. Ethical Considerations: Although the ethical aspects are addressed, adding a brief mention of any steps taken to minimize bias or stigmatization would further enhance transparency in research ethics.

**Conclusions**: Strengthen the conclusions by linking the findings more explicitly to potential policy responses or interventions. Mention any ongoing or future research that might address gaps left by this study. This would also help connect the study’s relevance to broader public and governmental policies.

Standardizing these sections will help align the paper more closely with scientific conventions and increase its clarity and impact.
