# Supplementary material for: The distribution of technology induced job loss: Evidence from a population-wide study in Norway
Source: PLoS One. 2025 Apr 15;20(4):e0321072. doi: 10.1371/journal.pone.0321072 (PMC11999129; doi:10.1371/journal.pone.0321072)
Supplement: Attachment — Submitted filename: Response letter.docx [file pone.0321072.s018.docx]

**PONE-D-23-25076R1 Response letter**

**General comment to both reviewers**

We sincerely thank the reviewers for taking their time to assess our study and for offering insightful comments and constructive feedback. Below, we address the comments and explain how we have taken them into account in the manuscripts. In cases where we reference our revised manuscript and have made additions to the existing text, the changes are highlighted in yellow.

**Note:** Page numbers and line numbers referred to in this letter are from the tracked changes version of the manuscript (page number in the middle of the bottom of each page).

**Reviewer #3:**

*1. Introduction
 - Clarification of research background: While the introduction already mentions the impact of technological changes on the labor market, further refinement of this background, particularly in the context of Norway, would be beneficial. For instance, specific Norwegian policies or trends in automation and globalization could be highlighted.*

**Response:**

We have added some more context, both related to the Norwegian economy and trends in automation, on page 3, line 53:

“Norway is a small, open economy with a highly educated workforce, extensive social safety net, and a compressed wage structure with centralized wage bargaining and strong labor unions. These factors have been argued to promote the adoption of technological innovations by industry [16]. Over recent decades, Norway has experienced both an increase in employment within high-skill occupations and a rise in automation through the use of industrial robots, reflecting broader trends observed in other developed economies [17, 18].”

*- Precision in stating research questions: In the introduction, it would be clearer to delineate the research questions, such as specifying which gender and socioeconomic disparities are to be investigated and how these disparities evolve over time.*

**Response:**

Based on this comment we have increased the precision of our research questions, on page 4, line 69 (changes highlighted):

“The study aimed to investigate how a widely used indicator of occupational automation risk is associated and aligned at the individual level with measures of socioeconomic status in a register based study of the full population of Norwegian 45-year-olds, and how this differs for men and women. Our specific objectives were to (i) examine and explain the development in sex-specific risk across birth cohorts, and (ii) to estimate the association with other risk factors of social marginalization, including educational attainment, income, family background and health measures.”
 *2. Literature Review
 - Exhaustiveness of literature citations: Although the paper references some relevant literature, incorporating more recent and authoritative sources to support the arguments, especially studies related to Norway or other European countries, would strengthen the review.*

**Response:**

We have added several relevant, recent and authoritative sources to support our argument.

The following was added in the introduction, [7] and [8] are highly authoritative authors within this field, [19] is a recent study from Norway, and [20] is a paper building a theoretical argument for the distributional effects of automation in a very prestigious academic journal.

7. Acemoglu, D., C. Lelarge, and P. Restrepo, Competing with Robots: Firm-Level Evidence from France. AEA Papers and Proceedings, 2020. 110: p. 383–88.

8. Acemoglu, D. and P. Restrepo, Robots and Jobs: Evidence from US Labor Markets. Journal of Political Economy, 2020. 128(6): p. 2188-2244.

19. Umblijs, J., P. Schone, and H. Finseraas, Automation and worker organisation. Applied Economics Letters, 2024: p. 1-5.

20. Moll, B., L. Rachel, and P. Restrepo, Uneven Growth: Automation's Impact on Income and Wealth Inequality. Econometrica, 2022. 90(6): p. 2645-2683.

*- Critical analysis of existing studies: When reviewing existing literature, a critical analysis of previous research should be conducted, identifying their limitations or research gaps, thereby clearly articulating the contribution of this study.*

**Response:**

We have added a clearer statement of our contribution in relation to the literature, on page 3, line 61:

“While previous studies primarily have focused on the negative impacts of automation on employment or inequality, less is known regarding the *distribution* of this risk across the population and to what extent we should expect it to dampen or amplify pre-existing socioeconomic differences. In particular, evidence on the development over time within subgroups and its association with other known risk factors of social marginalization, is scarce.”

*3. Methods
 - Detailed description of data: When describing the data sources and analysis sample, provide a more detailed account of the data acquisition process, processing methods, and measures taken to ensure data quality.*

**Response:**

We have added some more details about the data sources and the process related to getting access and ensuring data quality, on page 4, line 79:

“We used linked population-wide administrative register data, covering information from several national registries. The registers were linked by Statistics Norway and then deidentified before researchers were allowed access. The acquisition of the data was part of the data structure at the Center for Fertility at the Norwegian Institute of Public Health, where data sets from the different registers were linked and stored at a secure server hosted by the Services for sensitive data (TSD), University of Oslo.

The information included in the database was demographic information and family status (national population register), labor market history (employer and employee register), educational attainment (national educational database), and physician visits (Norway Control and Payment of Health Reimbursements database). The data were accessed by the researchers for research purposes on March 1st, 2020.”

*- Explanation of analytical methods: For the RTI index and other statistical methods employed, offer more explanations and theoretical rationale to help readers better understand the applicability and advantages of these methods.*

**Response:**

We have explained the RTI index in some more detail to help the reader understand why this concept is meaningful for this type of analysis, on page 6, line 113:

“To measure structural risk of automation at the occupation level, we use a routine task intensity (RTI) index suggested in Acemoglu and Autor (2011). The measure is a theoretically informed indicator of structural economic risk, classifying occupations based on the tasks performed in it. In particular, the index captures the extent to which an occupation is characterized by routine tasks in the cognitive, manual or interpersonal domain. This reflects an economic theory of automation predicting that routine tasks are more easily automated, an assumption that has received extensive empirical support in work employing the RTI index (Autor,2003). Measurement of the content of job tasks were retrieved from the O*NET database. This database consists of a highly detailed description of tasks performed in an occupation. From the large set of measurements in O*NET, Acemoglu and Autor (2011) suggests a subset of items which describe these dimensions. To summarise these items to a one-dimensional RTI measure for each occupation, we used the method suggested by Lewandowski et al. (2017) (see Supplementary material for details). Last, to ease interpretation, we standardized the RTI index distribution in the sample to be mean zero with a standard deviation of 1 (RTI z-score).”

*- Robustness checks: Although supplementary materials mention conducting robustness checks using different indicators, briefly mentioning these results in the main text would enhance the reliability of the conclusions.*

**Response:**

Robustness results are already noted both in the Methods section and in the results section:

Methods section: After presenting the RTI in the Methods section, since robustness concerns typically would be related to our measure of routine intensity, with the following text, on page 7, line 140:

“The main analysis in this paper is based on the 2003 version of the RTI – the first year of our study period. However, in the Supplementary material we also present all main results using both the FOI and the RTI based on 2019 O*Net-data to assess robustness of our conclusions against changes in task content over time. S1 Table shows that all these measures of structural change are highly correlated, and our main conclusions are therefore independent of the selected measure.”

Results section - gender specific trends:

In the results section in relation to gender-specific trends we have the following, on page 11, line 223:

“In the Supplementary material we also present the average risk score using the Frey-Osbourne index (S2 Fig), RTI 2019 z-score (S3 Fig), and not z-standardized RTI scores (S4 Fig). It should be noted that when presenting time trends with the FOI or raw RTI-score, the time trend is downward sloping, indicating that the overall risk of automation is falling during the study period. However, the decrease is smaller for men, causing an increasing gender gap.”

Results section - in relation to risk factors:

On page 11, line 235:

“See the Supplementary material for models using the Frey-Osbourne index (S5 Fig) and RTI 2019 z-score (S6 Fig).”

*4. Results
 - Clarity in presentation of results: When presenting study results, use figures or tables to visually display the disparities between gender and socioeconomic status more intuitively.*

**Response:**

Based on this comment, and a more concrete comment made by another referee, we have thoroughly revised all legends and captions to ensure figures are consistent, self-explanatory and intuitive. We think this has been a substantial improvement.

This comment did not provide specific guidance on which aspects of our tables and figures needed revising, but our assessment was that legends and captions needed substantial adjustments.

*5. Discussion and Conclusion
 - Comprehensive discussion: In the discussion section, consider a more comprehensive examination of other potential explanatory factors, such as cultural and policy environments, to eliminate other potential confounding factors.*

**Response:**

This paper presents a description of how an occupation level measure of automation risk is associated with individual-level measures of socioeconomic status. This gives an overview of whether automation risks would plausible exacerbate existing inequalities. This is not a causal study, as clearly indicated in the title. There are hence no confounding factors in the standard sense given this design: if we find that people with low education and income are more likely to work in high RTI occupations this is so even if the individuals involved should turn out to have other protective factors not available to us in our data.

We have clarified this in the Strength and weaknesses section by noting that our results only assess how occupational RTI is correlated with the specific socioeconomic indicators used in our study and that other protective factors or risk factors may show different associations, on page 16, line 351 (addition highlighted):

”Second, the scope of the study is limited to what can be quantitatively measured in administrative registers and may not capture other risk factors or protective factors that could show different associations to occupational RTI scores, for example important experiential aspects related to technological development that could be measured with qualitative methods.”

*- Clarity of conclusions: The conclusion should explicitly state the main findings, contributions, and implications for policymakers and researchers.*

**Response:**

We think the conclusion clearly presents our main findings. The comment gives no specific examples as to what issues the referee has with our conclusions and discussion or in what way they are too vague or could be improved. But, to become even more direct regarding policy implications, we have added the following, on page 16, line 359 (additions highlighted):

“...Given the increasing disparities identified in this study, policy makers should monitor the development in job market opportunities for various subgroups of the working-age population, and evaluate programs aimed at supporting individuals that are particularly negatively affected. For example, given the heightened risk faced by individuals with lower levels of education, policymakers should consider implementing targeted upskilling programs that equip these workers—especially younger individuals—with skills that are in high demand and less likely to be at risk of automation. Last, provided the recent rapid developments in generative AI, future research should also explore how the risk distribution is evolving over time across age-groups, industries and social risk factors.”

*- Future research directions: It would be beneficial to propose future research directions, such as further exploring differences across different age groups or industries, or studying the impact of technological changes on various social welfare indicators.*

**Response:**

We agree with this comment, and have added the following, on page 17, line 366:

“Last, provided the recent rapid developments in generative AI, future research should also explore how the risk distribution is evolving over time across age-groups, industries and social risk factors.”

**Reviewer #4:**

*1. Introduction: Ensure the introduction highlights the research gap more explicitly. This will better establish the context for the study and its relevance to the field. Furthermore, summarizing the hypothesis or key research questions can sharpen the focus.*

**Response:**

We agree that this aspect could be improved, and have taken following actions in response to your comment:

Research gap: we have added a clearer statement of our contribution in relation to the literature, on page 3, line 61:

“While previous studies primarily have focused on the negative impacts of automation on employment or inequality, less is known regarding the *distribution* of this risk across the population and to what extent we should expect it to dampen or amplify pre-existing socioeconomic differences. In particular, evidence on the development over time within subgroups and its association with other known risk factors of social marginalization, is scarce.”

Summarizing hypotheses: we added the following to the second paragraph of the introduction, on page 4, line 66:

“We hypothesize that men with lower education, who are more likely to be in routine-based occupations requiring fewer social skills, are at an increasingly higher risk of automation. Such a trend could exacerbate existing social inequalities [20].”

Key research question: we have rewritten research questions for increased precision, to the following, on page 4, line 69:

“The study aimed to investigate how a widely used indicator of occupational automation risk is associated and aligned at the individual level with measures of socioeconomic status in a register based study of the full population of Norwegian 45-year-olds, and how this differs for men and women. Our specific objectives were to (i) examine and explain the development in sex-specific risk across birth cohorts, and (ii) to estimate the association with other risk factors of social marginalization, including educational attainment, income, family background and health measures.”

*Methodology Clarification:
 1. Clearly specify the reasoning behind using the RTI index over other measures of automation risk like the Frey-Osborne Index, even though both are discussed. Include a brief comparison in the methodology section, not just in the results section, to improve clarity for readers.*

**Response:**

In response to this comment we specified the reasoning behind our choice with the following, on page 6, line 113:

“To measure structural risk of automation at the occupation level, we use the routine task intensity (RTI) index suggested in Acemoglu and Autor (2011)[21]. This approach to measuring routine intensity has been applied extensively in the labor economics literature on the impacts of skill based technological change, starting with the seminal contribution of Autor et al. (2003)[22].”

Then, later in the methodology section, we discuss briefly alternatives, on page 6, line 131:

“The RTI is one of two commonly used measures used to assess the risk of automation, with the other being the Frey-Osborne index (FOI) [22, 24]. The main difference between these measures is that the RTI is task-based – it assesses to what extent a job consists of tasks that could be automated – while the FOI is based on expert assessment of occupations. The measures are correlated, but RTI has a stronger theoretical foundation, allows for occupational scores to change with their task content [25], has done comparatively better in predictive tests [26], and has been used in an extensive literature on skill biased technological change [2, 22, 27-29] (see [30] and [31] for a description and discussion of the difference between these measures, and other alternative measures used in the literature).”

Our opinion is that this level of discussion is sufficient, and that more would decrease the readability of the paper. If the referee strongly disagrees, we are certainly willing to reconsider.

*2. Add more detail about how data access and linkage were done (e.g., anonymization procedures and encryption).*

**Response:**

We have added the following to better explain these aspects on page 4, line 79:

“We used linked population-wide administrative register data, covering information from several national registries. The registers were linked by Statistics Norway and then deidentified before researchers were allowed access. The acquisition of the data was part of the data structure at the Center for Fertility at the Norwegian Institute of Public Health, where data sets from the different registers were linked and stored at a secure server hosted by the Services for sensitive data (TSD), University of Oslo.

The information included in the database was demographic information and family status (national population register), labor market history (employer and employee register), educational attainment (national educational database), and physician visits (Norway Control and Payment of Health Reimbursements database). The data were accessed by the researchers for research purposes on March 1st, 2020.”

*Data Presentation:
 1. Ensure consistency in reporting the variables. The terms used for categories (e.g., education, income) should remain consistent throughout.*

**Response:**

Thank you for bringing this to our attention. In the revised manuscript we have revised labeling of education levels and income both in the manuscript and supplementary information to ensure consistency.

(Note, as mentioned on page 8, line 171, income variables are not reported in the table since these are divided in quintiles - it does not make sense to report its distribution, since it has been created by us.)

*2. Consider providing more detailed tables or visual aids in the results section for critical variables to enhance clarity.*

**Response:**

We have thoroughly revised legends, captions and axis labels to provide a much better guide to the figures - this was a substantial improvement.

*Discussion Section:
 1. Address the limitations more extensively, particularly focusing on the external validity of the findings outside of Norway.*

**Response:**

We have expanded the following to the limitations, on page 16, line 343:

“At the same time, our study has several weaknesses. First, the study has limited generalizability due to its focus on Norway, a country with unique characteristics related to gender roles, institutional environments, and industry structure. For example, Norway has an extensive welfare system that compensates 62.4 percent of the income in the case of job loss. Hence, the external validity of the results is limited, and likely most relevant for Northern European countries with similar institutions and social norms.”

*2. Expand on the implications of automation beyond just Norway to make the discussion more globally relevant. Including comparisons to other European countries or worldwide trends would enhance the broader significance of the findings.*

**Response:**

We have added the following, on page 16, line 348:

“At the same time, similar gender-based occupational patterns are likely to exist in diverse geographic contexts. Consequently, the widening gender gap in automation risk and its association with social risk factors are likely present in countries also far from Norway.“

*Figures and Tables:
 1. Ensure all figures and tables are fully labelled and include clear legends. Some figures (like those comparing gender over time) could benefit from a more detailed description in the figure caption to ensure they are standalone and clear to readers.*

**Response:**

Thank you for this important comment. We have thoroughly revised all legends, captions, and axis labels to ensure figures are consistent, self-explanatory and intuitive. We have also revised some figures to improve clarity, e.g. axis labels of Fig 1.

*2. Ethical Considerations: Although the ethical aspects are addressed, adding a brief mention of any steps taken to minimize bias or stigmatization would further enhance transparency in research ethics.*

**Response:**

We agree that this aspect could be improved, and have added the following, on page 10, line 206:

“To achieve this, we used neutral language and avoided portraying vulnerable groups in ways that might be perceived as burdensome. Additionally, we refrained from reporting on groups small enough to risk identifying individuals.“

*Conclusions: Strengthen the conclusions by linking the findings more explicitly to potential policy responses or interventions. Mention any ongoing or future research that might address gaps left by this study. This would also help connect the study’s relevance to broader public and governmental policies.*

**Response:**

We have added the following to the conclusions, on page 16, line 359 (additions highlighted):

“...Given the increasing disparities identified in this study, policy makers should monitor the development in job market opportunities for various subgroups of the working-age population, and evaluate programs aimed at supporting individuals that are particularly negatively affected. For example, given the heightened risk faced by individuals with lower levels of education, policymakers should consider implementing targeted upskilling programs that equip these workers—especially younger individuals—with skills that are in high demand and less likely to be at risk of automation. Last, provided the recent rapid developments in generative AI, future research should also explore how the risk distribution is evolving over time across age-groups, industries and social risk factors.”
