# Supplementary material for: The distribution of technology induced job loss: Evidence from a population-wide study in Norway
Source: PLoS One. 2025 Apr 15;20(4):e0321072. doi: 10.1371/journal.pone.0321072 (PMC11999129; doi:10.1371/journal.pone.0321072)
Supplement: Attachment — Submitted filename: PONE-D-23-25076_R2_reviewer (1).docx [file pone.0321072.s017.docx]

**PLOS ONE**

**The Distribution of Technology Induced Job Loss: Evidence from a Population-wide Study in Norway**

--Manuscript Draft--

| **Manuscript Number:** | PONE-D-23-25076R2 |
| --- | --- |
| **Article Type:** | Research Article |
| **Full Title:** | The Distribution of Technology Induced Job Loss: Evidence from a Population-wide Study in Norway |
| **Short Title:** | The Distribution of Technology Induced Job Loss |
| **Corresponding Author:** | Bjørn-Atle Reme  Norwegian Institute of Public Health: Folkehelseinstituttet OSLO, NORWAY |
| **Keywords:** | Automation; social inequality; gender; labor market |
| **Abstract:** | Globalization and automation are leading to skill-biased structural changes in the labor market, resulting in the polarization of employment opportunities. There is increasing concern that the relative decline of occupations characterized by routine cognitive and physical tasks is contributing to growing earnings- and gender inequality. Using comprehensive individual-level data from Norway, our analysis reveals significant and growing gender disparities in the routine intensity of occupations. Additionally, we show that the gender disparities are particularly strong among individuals with low socioeconomic status, and that the main contributor to the growing gender disparities is the increasing gender difference in educational attainment. |
| **Order of Authors:** | Bjørn-Atle Reme |
|  | Ole Røgeberg |
|  | Bernt Bratsberg |
|  | Jonathan Wörn |
|  | Vegard F. Skirbekk |
| **Response to Reviewers:** | See separate attachment with response to reviewers letter. |
| **Additional Information:** |  |
| **Question** | **Response** |
| **Financial Disclosure**  Enter a financial disclosure statement that describes the sources of funding for the work included in this submission. Review the [submission guidelines](http://journals.plos.org/plosone/s/submission-guidelines#loc-financial-disclosure-statement) for detailed requirements. View published research articles from [*PLOS ONE*](http://journals.plos.org/plosone/) for specific examples.  This statement is required for submission and **will appear in the published article** if the submission is accepted. Please make sure it is accurate. | This work was financed by the Research Council of Norway through its Centres of Excellence funding scheme (project number 262700) and the project DIMJOB (project number 296297). |

| **Unfunded studies**  Enter: *The author(s) received no specific funding for this work.*  **Funded studies**  Enter a statement with the following details:   - Initials of the authors who received each award - Grant numbers awarded to each author - The full name of each funder - URL of each funder website - Did the sponsors or funders play any role in the study design, data collection and analysis, decision to publish, or preparation of the manuscript? - **NO** - Include this sentence at the end of your statement: *The funders had no role in study design, data collection and analysis, decision to publish, or preparation of the manuscript.* - **YES** - Specify the role(s) played. |  |
| --- | --- |
| * typeset |  |
| **Competing Interests**  Use the instructions below to enter a competing interest statement for this submission. On behalf of all authors, disclose any [competing interests](http://journals.plos.org/plosone/s/competing-interests) that could be perceived to bias this work—acknowledging all financial support and any other relevant financial or non- financial competing interests.  This statement is required for submission and **will appear in the published article** if the submission is accepted. Please make sure it is accurate and that any funding sources listed in your Funding Information later in the submission form are also declared in your Financial Disclosure statement.  View published research articles from  [*PLOS ONE*](http://journals.plos.org/plosone/) for specific examples. | We declare no competing interests. |

| **NO authors have competing interests**  Enter: *The authors have declared that no competing interests exist.*  **Authors with competing interests**  Enter competing interest details beginning with this statement:  *I have read the journal's policy and the authors of this manuscript have the following competing interests: [insert competing interests here]* |  |
| --- | --- |
| * typeset |  |
| **Ethics Statement**  Enter an ethics statement for this submission. This statement is required if the study involved:   - Human participants - Human specimens or tissue - Vertebrate animals or cephalopods - Vertebrate embryos or tissues - Field research   Write "N/A" if the submission does not require an ethics statement.  General guidance is provided below. Consult the [submission guidelines](http://journals.plos.org/plosone/s/submission-guidelines#loc-guidelines-for-specific-study-types) for detailed instructions. **Make sure that all information entered here is included in the Methods section of the manuscript.** | The ethical approval for this study was given by The Ethics Committee of South-East Norway (#2018/434). On the basis of Section 35 of the Health Research Act, participant consent was waived by the Comittee. Data from the different registers were linked by certified researchers who only had access to encrypted personal ID-variable. |

| **Format for specific study types**  **Human Subject Research (involving human participants and/or tissue)**   - Give the name of the institutional review board or ethics committee that approved the study - Include the approval number and/or a statement indicating approval of this research - Indicate the form of consent obtained (written/oral) or the reason that consent was not obtained (e.g. the data were analyzed anonymously)   **Animal Research (involving vertebrate animals, embryos or tissues)**   - Provide the name of the Institutional Animal Care and Use Committee (IACUC) or other relevant ethics board that reviewed the study protocol, and indicate whether they approved this research or granted a formal waiver of ethical approval - Include an approval number if one was obtained - If the study involved *non-human primates*, add *additional details* about animal welfare and steps taken to ameliorate suffering - If anesthesia, euthanasia, or any kind of animal sacrifice is part of the study, include briefly which substances and/or methods were applied   **Field Research**  Include the following details if this study involves the collection of plant, animal, or other materials from a natural setting:   - Field permit number - Name of the institution or relevant body that granted permission |  |
| --- | --- |
|  |  |
| **Data Availability**  Authors are required to make all data underlying the findings described fully available, without restriction, and from the time of publication. PLOS allows rare exceptions to address legal and ethical concerns. See the [PLOS Data Policy](http://journals.plos.org/plosone/s/data-availability) and [FAQ](http://journals.plos.org/plosone/s/data-availability#loc-faqs-for-data-policy) for detailed information. | No - some restrictions will apply |

| A Data Availability Statement describing where the data can be found is required at submission. Your answers to this question constitute the Data Availability Statement and **will be published in the article**, if accepted. |  |
| --- | --- |
| **Important:** Stating ‘data available on request from the author’ is not sufficient. If your data are only available upon request, select ‘No’ for the first question and explain your exceptional situation in the text box. |  |
| Do the authors confirm that all data underlying the findings described in their manuscript are fully available without restriction? |  |
| **Describe where the data may be found in full sentences. If you are copying our sample text, replace any instances of XXX with the appropriate details.**   - If the data are **held or will be held in a public repository**, include URLs, accession numbers or DOIs. If this information will only be available after acceptance, indicate this by ticking the box below. For example: *All XXX files are available from the XXX database (accession number(s) XXX, XXX.*). - If the data are all contained **within the manuscript and/or Supporting Information files**, enter the following: *All relevant data are within the manuscript and its Supporting Information files.* - If neither of these applies but you are able to provide **details of access elsewhere**, with or without limitations, please do so. For example:   *Data cannot be shared publicly because of [XXX]. Data are available from the XXX Institutional Data Access / Ethics Committee (contact via XXX) for researchers who meet the criteria for access to confidential data.*  *The data underlying the results presented in the study are available from (include the name of the third party* | The data used in this study encompasses educational outcomes, income, employment records, health records and demographic information for entire cohorts of the Norwegian population. These data were made available on loan for research purposes. Other researchers may apply for access to the same sources - see “helsedata.no/en” and [“https://www.ssb.no/en/data-til-forskning/utlan-av-data-til-forskere”.](http://www.ssb.no/en/data-til-forskning/utlan-av-data-til-forskere) |

| *and contact information or URL).*   - This text is appropriate if the data are owned by a third party and authors do not have permission to share the data.   * typeset |  |
| --- | --- |
| Additional data availability information: |  |

Manuscript	[Click here to access/download;Manuscript;Manuscript.docx](https://www2.cloud.editorialmanager.com/pone/download.aspx?id=38317231&guid=c6521422-a241-47bf-bc7b-fed971f0485c&scheme=1)[<https://www2.cloud.editorialmanager.com/pone/download.aspx?id=38317231&guid=c6521422-a241-47bf-bc7b-fed971f0485c&scheme=1>](https://www2.cloud.editorialmanager.com/pone/download.aspx?id=38317231&guid=c6521422-a241-47bf-bc7b-fed971f0485c&scheme=1)

| 1 | **The Distribution of Technology Induced Job Loss:** |  | |
| --- | --- | --- | --- |
| 2 | **Evidence from a Population-wide Study in Norway** |  |  |
| 3 |  |  |  |
| 4 |  |  |  |
| 5 |  |  |  |
| 6 | Bjørn-Atle Reme^1,*^, Ole Røgeberg^2^, Jonathan Wörn^1^, Bernt Bratsberg^2^, | Vegard | F. |
| 7 | Skirbekk^1^ |  |  |
| 8 |  |  |  |
| 9 |  |  |  |
| 10 | ^1^Norwegian Institute of Public Health, Oslo, Norway |  |  |
| 11 | ^2^Frisch Center, Oslo, Norway |  |  |
| 12 |  |  |  |
| 13 | * Corresponding author |  |  |
| 14 | E-mail: [bjorn-atle.reme@fhi.no](mailto:bjorn-atle.reme@fhi.no) |  |  |
| 15 |  |  |  |
| 16 |  |  |  |
| 17 |  |  |  |
| 18 |  |  |  |

# 19 Abstract

20 Globalization and automation are leading to skill-biased structural changes in the
21 labor market, resulting in the polarization of employment opportunities. There is increasing
22 concern that the relative decline of occupations characterized by routine cognitive and
23 physical tasks is contributing to growing earnings- and gender inequality. Using
24 comprehensive individual-level data from Norway, our analysis reveals significant and
25 growing gender disparities in the routine intensity of occupations. Additionally, we show
26 that the gender disparities are particularly strong among individuals with low socioeconomic
27 status, and that the main contributor to the growing gender disparities is the increasing
28 gender difference in educational attainment.

# 29 Introduction

30 In recent decades, skill-biased technological change has allowed firms to automate
31 and offshore routine-intensive tasks, leading to an erosion of low skilled and mid-level jobs
32 and increasing returns to education [1-4]. These trends are expected to continue, and
33 according to the OECD, approximately 14% of jobs are at risk of automation [5]. While the
34 disappearance of routine-intensive occupations may not necessarily result in an increase in
35 long-term unemployment [6], it does result in a shift in the labor market structure [7, 8]. In
36 fact, a recent OECD policy-brief found that countries with higher investments in robotics
37 experienced *higher* employment growth [9], but also heightened job instability among low-
38 educated workers, typically concentrated within occupations with a high risk of automation.
39 This raises the question of whether a continuation of these trends will serve to amplify or
40 dampen pre-existing inequalities [10, 11].
41 Several studies find that less-educated men is a group that has experienced stagnation
42 or declines in both wage level and labor market participation [12, 13]. This development has
43 coincided with a decline in marriage rates, fertility, and health outcomes [13-15]. While the
44 reasons behind these developments are largely unknown, it has stirred debates among policy
45 makers and academics as to the role of technological development.
46 Norway is a small, open economy with a highly educated workforce, extensive social
47 safety net, and a compressed wage structure with centralized wage bargaining and strong
48 labor unions. These factors have been argued to promote the adoption of technological
49 innovations by industry [16]. Over recent decades, Norway has experienced both an increase
50 in employment within high-skill occupations and a rise in automation through the use of
51 industrial robots, reflecting broader trends observed in other developed economies [17, 18].
52 Recent evidence from Norway suggests that while automation has negative impacts on
53 employment and wages, these effects are, to some extent, mitigated by labor unions [19].
54 While previous studies primarily have focused on the negative impacts of automation on
55 employment or inequality, less is known regarding the distribution of this risk across the
56 population and to what extent we should expect it to dampen or amplify pre-existing
57 socioeconomic differences. In particular, evidence on the development over time within
58 subgroups and its association with other known risk factors of social marginalization, is
59 scarce. We hypothesize that men with lower education, who are more likely to be in routine-
60 based occupations requiring fewer social skills, are at an increasingly higher risk of
61 automation. Such a trend could exacerbate existing social inequalities [20].
62 The study aimed to investigate how a widely used indicator of occupational
63 automation risk is associated and aligned at the individual level with measures of
64 socioeconomic status in a register based study of the full population of Norwegian 45-year-
65 olds, and how this differs for men and women. Our specific objectives were to (i) examine
66 and explain the development in gender-specific risk across birth cohorts, and (ii) to estimate
67 the association with other risk factors of social marginalization, including educational
68 attainment, income, family background and health measures.

# 69 Methods

## 70 Data and analytical sample

71 We used linked population-wide administrative register data, covering information
72 from several national registries. The registers were linked by Statistics Norway and then
73 deidentified before researchers were allowed access. The acquisition of the data was part of
74 the data structure at the Center for Fertility at the Norwegian Institute of Public Health, where
75 data sets from the different registers were linked and stored at a secure server hosted by the
76 Services for sensitive data (TSD), University of Oslo.
77 The information included in the database was demographic information and family
78 status (national population register), labor market history (employer and employee register),
79 educational attainment (national educational database), and physician visits (Norway
80 Control and Payment of Health Reimbursements database). The data were accessed by the
81 researchers for research purposes on March 1st, 2020.
82 Our sample population was all individuals born 1958-1973 who were employed in
83 the year they turned 45 (N = 900,559) and living in Norway. Since there are considerable
84 differences in early-career trajectories across education levels, we measured the structural
85 risk of automation of the occupation held mid-career, at age 45. Our study period covers
86 2003-2018.
87 To measure structural risk of automation at the occupation level, we use the routine
88 task intensity (RTI) index suggested in Acemoglu and Autor (2011)[21]. This approach to
89 measuring routine intensity has been applied extensively in the labor economics literature
90 on the impacts of skill based technological change, starting with the seminal contribution of
91 Autor et al. (2003)[22]. The measure is a theoretically informed indicator of structural
92 economic risk, classifying occupations based on the tasks performed in it. In particular, the
93 index captures the extent to which an occupation is characterized by routine tasks in the
94 cognitive, manual or interpersonal domain. This reflects an economic theory of automation
95 predicting that routine tasks are more easily automated, an assumption that has received
96 extensive empirical support in work employing the RTI index [22]. Measurement of the
97 content of job tasks were retrieved from the O*NET database. This database consists of a
98 highly detailed description of tasks performed in an occupation. From the large set of
99 measurements in O*NET, Acemoglu and Autor (2011) suggests a subset of items which
100 describe these dimensions. To summarize these items to a one-dimensional RTI measure for
101 each occupation, we used the method suggested by Lewandowski et al. (2017) (see
102 Supplementary material for details) [23]. Last, to ease interpretation, we standardized the
103 RTI score distribution in the sample to be mean zero with a standard deviation of 1 (RTI z-
104 score).
105 The RTI is one of two commonly used measures used to assess the risk of automation,
106 with the other being the Frey-Osborne index (FOI) [22, 24]. The main difference between
107 these measures is that the RTI is task-based – it assesses to what extent a job consists of tasks
108 that could be automated – while the FOI is based on expert assessment of occupations. The
109 measures are correlated, but RTI has a stronger theoretical foundation, allows for
110 occupational scores to change with their task content [25], has done comparatively better in
111 predictive tests [26], and has been used in an extensive literature on skill biased technological
112 change [2, 22, 27-29] (see [30] and [31] for a description and discussion of the difference
113 between these measures, and other alternative measures used in the literature).
114 The main analysis in this paper is based on the 2003 version of the RTI – the first
115 year of our study period. However, in the Supplementary material we also present all main
116 results using both the FOI and the RTI based on 2019 O*Net-data to assess robustness of
117 our conclusions against changes in task content over time. S1 Table shows that all these
118 measures of structural change are highly correlated, and our main conclusions are therefore
119 independent of the selected measure.
120 Other measures used as covariates for stratification in the analysis were the following:
121 Education: The highest level of completed education, available from the National Education
122 Database. There are 9 levels within the ISCED classification, where level 9 is “unknown”.
123 In the analysis we either use 4 categories: primary (1-2), secondary (3-5), 1–4-year university
124 education (6) and master and higher university education (7-8), or dichotomize by whether
125 or not the individual had university level education or not, i.e. 1-5 vs 6-8, referred to as “low”
126 and “high” education, respectively.
127 Own income: Own income was retrieved from the income tax register. We calculated
128 birthyear- and gender-stratified income quintiles. In the main part of the analysis, we use a
129 binary variable indicating whether the individual belonged to the lowest income quintile.
130 Father income: Father’s income was retrieved from the income tax register. We calculated
131 the father’s income quintile at age 40, stratified on his birth cohort. In the main part of the
132 analysis, we use a binary variable indicating whether the father belonged to the lowest
133 income quintile.
134 Marital status: Marital status was retrieved from the official marriage register. This contains
135 complete records of marriages in Norway since 1974. For the analysis we used a binary
136 indicator for whether the individual was registered as married at age 45.
137 Childlessness: Using the population register we created a binary indicator for whether the
138 individual was childless at age 45.
139 Musculoskeletal/psychological problems: We used the register of primary care utilization
140 (KUHR) to create a binary indicator for whether the individual had visited primary health
141 care (general practitioner) for musculoskeletal (L-chapter symptoms or diagnoses) and/or
142 psychological (P-chapter symptoms or diagnoses) problems during the year when being 45-
143 years old. These two chapters were chosen, as they are the most common reasons for both
144 health problems and sick leave among 45-year-olds in Norway.
145 Table 1 provides summary statistics across covariates, excluding covariates based on
146 creating groups based on the underlying distribution (quintiles for own income and parental
147 income).
148 **Table 1. Summary statistic for covariates**

|  | **Covariate** | **Proportion** |
| --- | --- | --- |
|  | Female | 49% |
|  | Childless | 15% |
|  | Married | 57% |
|  | Primary education | 25% |
|  | Secondary education | 36% |
|  | Low university education (Bachelor’s) | 29% |
|  | High university (Master’s or or PhD) | 10% |
|  | Visit to doctor for psychological problem | 9% |
|  | Visit to doctor for musculoskeletal problem | 22% |
|  | Number of observations (N) | 900,559 |
| 149 |  |  |

## 150 Empirical strategy and statistical analysis

151 The aim was to examine the relationship between structural risk of automation at the
152 occupation level and indicators of socioeconomic status. All individuals in our sample were
153 assigned an RTI score based on the occupation they had at age 45. Our aim was then to
154 assess how this occupational risk score was associated with other measures of socioeconomic
155 risk factors. To assess the extent of structural shifts in the labor market throughout our
156 sample period we estimated the year- and gender-specific average RTI z-score for 45-year-
157 olds.
158 We then examined how structural risk of automation (RTI z-score) was associated
159 with our measures of socioeconomic status and health: Using simple and multivariate
160 regression models separately for each gender, we assess the extent to which RTI score is
161 predicted by the other measures, with birth cohort dummies used to control for potential time
162 trends.
163 Next, we performed an attribution analysis where we estimated how much of the
164 increase in automation risk gender gap can be explained by the various covariates included
165 in the regression models.
166 Finally, to examine the overall variation in occupational risk scores in most current
167 population of 45-year-olds in our data - birth cohort 1973 - we predicted the RTI z-score of
168 men and women for each observed combination of covariate values and plotted the ordered
169 estimates in a bubble scatter plot where the bubble size reflects the size of the covariate
170 group. To examine how these patterns have evolved over time, we also reproduced the plot
171 for the first birth cohort in our data (born in 1958).

## 172 Ethical considerations

173 The ethical approval for this study was given by The Ethics Committee of South-East
174 Norway (#2018/434). Based on Section 35 of the Health Research Act, participant consent
175 was waived by the Committee. Data from the different registers were linked by certified
176 researchers who only had access to encrypted personal ID-variable. Extensive measures
177 were taken to maintain the security and confidential handling of the research data.
178 Throughout the process of analyzing data and presenting findings, we are committed to
179 preventing stigmatization and to upholding ethical principles of honesty and transparency in
180 reporting our findings. To achieve this, we used neutral language and avoided portraying
181 vulnerable groups in ways that might be perceived as burdensome. Additionally, we
182 refrained from reporting on groups small enough to risk identifying individuals.

# 183 Results

## 184 Gender specific time trends in routine intensity

185 Our data covers the 15-year period 2003 to 2018. We first examined the development
186 in routine intensity across this period, separately for each gender (Figure 1). In the
187 supplementary materials we also present the development in RTI score for each of the
188 covariates used in the analysis (S1 Fig).

189

#### 190 Fig 1. The yearly average routine intensity index among 45-year-olds by gender. The

191 figure shows the yearly average RTI z-score, stratified by year, for 45-year-olds (birth
192 cohorts 1958 to 1973) by gender. The 95% confidence intervals are indicated by the error
193 bars. See S2 Table in the Supplementary Material for a corresponding table.
194 While there already was a substantial gender difference in standardized routine

195	intensity in 2003 - the score was 0.13 [95% CI, 0.12-0.14] for men and -0.13 [95% CI, (-

196 0.14)-(-0.12)] for women - the difference gradually increased up until 2018, to 0.18 [95%
197 CI, 0.17-0.19] for men and -0.20 [95% CI, (-0.21)-(-0.19)] for women. In the Supplementary
198 material we also present the average risk score using the Frey-Osbourne index (S2 Fig), RTI
199 2019 z-score (S3 Fig), and not z-standardized RTI scores (S4 Fig). It should be noted that
200 when presenting time trends with the FOI or raw RTI-score, the time trend is downward
201 sloping, indicating that the overall risk of automation is falling during the study period.
202 However, the decrease is smaller for men, causing an increasing gender gap.

## 203 The association between structural risk and socioeconomic status

204 In bivariate analyses, RTI z-score was most strongly associated with low education
205 (education below university level) with coefficients of 1.11 (p < 0.001) for women and 0.82
206 for men (p < 0.001) (Figure 2A). Regarding gender, the association between RTI z-score and
207 socioeconomic risk factors were higher among men for almost all socioeconomic indicators
208 assessed. The exceptions were females who were childless or with a psychological diagnosis
209 from their physician. See the Supplementary material for models using the Frey-Osbourne
210 index (S5 Fig) and RTI 2019 z-score (S6 Fig).

211

212 **Figure 2. Routine intensity risk factors by gender.** The figure displays regression
213 coefficients from (A) bivariate and (B) multivariate regression models, with the RTI z-score
214 as the outcome variable for all 45-year-olds between 2003 and 2018, separately by gender.
215 The 95% confidence intervals are indicated by the error bars. Detailed regression tables are
216 provided in the Supplementary Material (S2 Table).

## 217 Explaining the increasing gender gap – attribution analysis

218 The gender differences in regression coefficients shown in Figure 2 could explain a
219 gender gap in the RTI. However, to understand why this gap has increased over time, we
220 also need to consider gender-specific changes in the prevalence of relevant risk factors. To
221 explore this further, we conduct an attribution analysis to assess how the gender-specific
222 development of each risk factor contributes to the overall change in the gap. This analysis
223 was carried out in two steps. First, the gender-specific change in prevalence over the period
224 2003 to 2018 was calculated (Figure 3A). Second, to estimate the contribution, we multiplied
225 these changes by the regression coefficients from the multivariate model (see Figure 2B) to
226 estimate the relative impact of each risk factor to the evolving gender-specific risk of
227 automation. These results are shown in Figure 3B.

228

#### 229 Figure 3. Attribution analysis of routine intensity risk factors among 45-year-olds in

230 **2003 and 2018, by gender.** (A) Share of 45-year-olds in 2003 and 2018 exposed to various
231 risk factors. (B) Estimated impact of changes in exposure, calculated by multiplying the
232 change in share (from Panel A) by the corresponding regression coefficient for each
233 characteristic, based on a multivariate regression of the RTI z-score on all risk factors (see
234 Figure 2B).
235 The largest change in risk factor exposure was related to an increase in higher
236 education (and correspondingly a decline in the share with low education; Figure 3A),
237 especially among women (from 33 to 58 percent). There was also a substantial increase in
238 the share of the sample that were unmarried, both among men (from 37 to 49 percent) and
239 women (35 to 47 percent). Regarding the impact of the change in exposure on RTI z-score,
240 i.e., when combining the strength of association (Figure 2B) with the change in exposure
241 (Figure 3A), the strongest impact was found for education with an estimated impact on the
242 RTI z-score of -0.117 (95% CI (-0.116)-(-0.118)) for men and -0.197 (95% CI (-0.195)-(-
243 0.198)) for women.

## 244 Distribution of structural risk across population groups

245 The estimated RTI z-scores from a multivariate model applied to the 2018 cohort of
246 45 years old individuals (birth cohort 1973) showed substantial variation across
247 combinations of risk factors (Figure 3). While the predicted RTI z-scores were -0.75 [95%
248 CI (-0.78)-(-0.72)] and -0.80 [95% CI (-0.83)-(-0.77) ] among highly educated married men
249 and women with children and from high SES families, the corresponding RTI z-scores were
250 0.82 [95% CI 0.69-0.95] and 0.37 [95% CI 0.17-0.58] among less educated unmarried men
251 and women without children from low SES families (see S3 Table and S4 Table for
252 corresponding tables). To examine how this distribution has developed over time, we also
253 estimated this model for the 2003 cohort (1958 birth cohort). The comparison across cohorts
254 reveals that while the gender difference in automation risk declined among highly educated
255 individuals, it has remained substantial among those of lower education. Hence, considering
256 the increasing share of women taking higher education, it supports our finding that the
257 increasing gender gap is due to a large shift in the share of women with university education
258 (S7 Fig).

259

#### 260 Figure 4. Predicted RTI z-scores from multivariate regression by gender. The figure

261 presents predicted RTI z-scores based on gender-specific models estimated for the 1973 birth
262 cohort (i.e., those aged 45 in 2018). The explanatory variables were indicators for educational
263 level (Primary educ = primary education; Secondary educ = secondary education; Low uni =
264 lower university (Bachelor’s degree); High uni = higher university (Master’s degree or
265 higher)), indicators for childlessness (Childless), marital status (Married), and whether the
266 father’s average income rank between ages 40 and 50 was within the lowest quintile among
267 men of the same age group (Low parental income). See Supplementary Material (S3 Table
268 and S4 Table) for detailed tables and the average RTI z-scores for each group.

# 269 Discussion

270 Our study has four main findings. First, the risk of automation was higher among
271 men than women. This difference increased from 2003 to 2018. Second, the risk of
272 automation was higher among individuals with low socioeconomic status and less social
273 support in the form of a partner and children. Third, the main reason for the increasing gender
274 gap was the growing gender differences in educational attainment. In summary, our study
275 suggests that automation may exacerbate existing social inequalities and create a more
276 unstable job market situation for men that may already struggle financially and socially. And,
277 if disparities in economic and social opportunity are allowed to grow without mitigating
278 policies, they have the potential to create social unrest and political polarization. At the same
279 time, the nature of technological development changes rapidly, as evidenced by the
280 introduction of large language models and multimodal models in recent years. These new
281 developments may have further increased the scope of automation, potentially increasing the
282 scope of routine tasks to also cover language-based tasks such as report writing and analysis.
283 Gender disparities in the risk of automation have also been found in other studies
284 covering several European countries [31]. Interestingly, whether men or women are found
285 to be at the higher risk of automation varies across countries, with the Nordic countries
286 having a higher risk among men. There could be several reasons for such differences across
287 countries, for example related to the Nordic countries having strong gender equality norms,
288 high growth in the education level among women, and lower levels of female part-time work.
289 Moreover, our study covers prime-aged individuals, and the risk likely varies substantially
290 across age. The importance of age is documented in a recent study assessing the causal
291 impact of robot- and information technology across several European countries. While the
292 impact of these technologies on employment were more negative among older women,
293 compared to older men, the impact in prime age (age 30-49) was considerably more negative
294 for men than for women [32].
295 We have found that the growing gender gap in the risk of automation can be
296 attributed, in large part, to the increasing levels of education among women. It is interesting
297 to note that this gap in education continues to widen, and Statistics Norway reported in 2019
298 that in many municipalities the share of highly educated women is more than twice that of
299 men [33].
300 As these developments are likely to continue, it is important for policy makers to
301 understand their implications and carefully consider the best ways to address and mitigate
302 the negative impacts.

## 303 Strengths and weaknesses

304 The main strength of our study is that it covers the full population of employed 45-
305 year-old individuals in Norway over a 15-year period across several important characteristics
306 related to health, financial position, education, and family formation. Hence, in contrast to
307 most other studies on this topic which uses survey data, our study does not suffer from
308 selection bias. At the same time, our study has several weaknesses. First, the study has
309 limited generalizability due to its focus on Norway, a country with unique characteristics
310 related to gender roles, institutional environments, and industry structure. For example,
311 Norway has an extensive welfare system that compensates 62.4 percent of the income in the
312 case of job loss. Hence, the external validity of the results is limited, and likely most relevant
313 for Northern European countries with similar institutions and social norms. At the same time,
314 similar gender-based occupational patterns are likely to exist in diverse geographic contexts.
315 Consequently, the widening gender gap in automation risk and its association with social
316 risk factors are likely present in countries also far from Norway. Second, the scope of the
317 study is limited to what can be quantitatively measured in administrative registers and may
318 not capture important experiential aspects related to technological development that could
319 be measured with qualitative methods.

# 320 Conclusion

321 The gender difference in the risk of automation has been increasing, with a particular
322 high risk among lower educated men with fewer family ties. This potentially has significant
323 and far-reaching negative impacts on individuals and society as it could exacerbate the
324 concentration of economic, social and health exclusion in the coming decades. Given the
325 increasing disparities identified in this study, policy makers should monitor the development
326 in job market opportunities for various subgroups of the working-age population, and
327 evaluate programs aimed at supporting individuals that are particularly negatively affected.
328 For example, given the heightened risk faced by individuals with lower levels of education,
329 policymakers should consider implementing targeted upskilling programs that equip these
330 workers—especially younger individuals—with skills that are in high demand and less likely
331 to be at risk of automation. Last, provided the recent rapid developments in generative AI,
332 future research should also explore how the risk distribution is evolving over time across
333 age-groups, industries and social risk factors.

334

# 335 Acknowledgements

336 **Authors' contributions**
337 O.R. and V.S. conceptualized the idea. B-A.R. and O.R. wrote the main manuscript. B-A.R.
338 were responsible for the statistical analysis. All authors contributed to designing the research
339 and the empirical strategy. All authors reviewed the manuscript.

340

341 **Availability of data and materials**
342 The data used in this study compriseseducational outcomes, income, employment records,
343 health records and demographic information for entire cohorts of the Norwegian population.
344 These data were made available on loan for research purposes. Other researchers may apply
345 for access to the same sources - see “helsedata.no/en” and “https:/[/www.ssb.no/en/data-til-](http://www.ssb.no/en/data-til-)
346 forskning/utlan-av-data-til-forskere”.

# 347 References

348 1.	Autor, D.H. and D. Dorn, *The Growth of Low-Skill Service Jobs and the Polarization of*
349 *the US Labor Market.* American Economic Review, 2013. **103**(5): p. 1553-97.
350 2.	Goos, M., et al., *Job Polarization in Europe.* American Economic Review, 2009. **99**: p.

351	58-63.

352 3.	Goos, M., A. Manning, and A. Salomons, *Explaining Job Polarization: Routine-Biased*
353 *Technological Change and Offshoring.* The American Economic Review, 2014. **104**.
354 4.	Graetz, G. and G. Michaels, *Robots at Work.* The Review of Economics and Statistics,

355	2018. **100**(5): p. 753-768.

356 5.	Nedelkoska, L. and G. Quintini, *Automation, skills use and training.* 2018.
357 6.	Genz, S., et al., *How Do Workers Adjust When Firms Adopt New Technologies?* 2021.
358 7.	Acemoglu, D., C. Lelarge, and P. Restrepo, *Competing with Robots: Firm-Level Evidence*
359 *from France.* AEA Papers and Proceedings, 2020. **110**: p. 383–88.
360 8.	Acemoglu, D. and P. Restrepo, *Robots and Jobs: Evidence from US Labor Markets.*
361 Journal of Political Economy, 2020. **128**(6): p. 2188-2244.
362 9.	Gregory, T., A. Salomons, and U. Zierahn, *Racing With or Against the Machine?*
363 *Evidence on the Role of Trade in Europe.* Journal of the European Economic
364 Association, 2022. **20**(2): p. 869-906.
365 10.	Bessen, J., *Automation and jobs: when technology boosts employment*.* Economic

366	Policy, 2019. **34**(100): p. 589-626.

367 11.	Schmidpeter, B. and R. Winter-Ebmer, *Automation, unemployment, and the role of*
368 *labor market training.* European Economic Review, 2021. **137**: p. 103808.
369 12.	Binder, A. and J. Bound, *The Declining Labor Market Prospects of Less-Educated Men.*
370 Journal of Economic Perspectives, 2019. **33**: p. 163-190.
371 13.	Bratsberg, B., A. Kotsadam, and S. Walther, *Male Fertility: Facts, Distribution and*
372 *Drivers of Inequality.* SSRN Electronic Journal, 2021.
373 14.	Lundberg, S., R. Pollak, and J. Stearns, *Family Inequality: Diverging Patterns in*
374 *Marriage, Cohabitation, and Childbearing †.* Journal of Economic Perspectives, 2016.
375 **30**: p. 79-102.
376 15.	Case, A. and A. Deaton, *Rising Morbidity and Mortality in Midlife Among White Non-*
377 *Hispanic Americans in the 21st Century.* Proceedings of the National Academy of
378 Sciences, 2015. **112**: p. 201518393.
379 16.	Barth, E., K.O. Moene, and F. Willumsen, *The Scandinavian model—An interpretation.*
380 Journal of Public Economics, 2014. **117**: p. 60-72.
381 17.	Jurkat, A., R. Klump, and F. Schneider, *Tracking the Rise of Robots: A Survey of the IFR*
382 *Database and its Applications*. 2021.
383 18.	Alasoini, T., et al., *Digital Transformations of Traditional Work in the Nordic Countries*
384 *- Edited by Bertil Rolandsson*. 2020.
385 19.	Umblijs, J., P. Schone, and H. Finseraas, *Automation and worker organisation.* Applied
386 Economics Letters, 2024: p. 1-5.
387 20.	Moll, B., L. Rachel, and P. Restrepo, *Uneven Growth: Automation's Impact on Income*
388 *and Wealth Inequality.* Econometrica, 2022. **90**(6): p. 2645-2683.
389 21.	Acemoglu, D. and D. Autor, *Chapter 12 - Skills, Tasks and Technologies: Implications*
390 *for Employment and Earnings**We thank Amir Kermani for outstanding research*
391 *assistance and Melanie Wasserman for persistent, meticulous and ingenious work on*
392 *all aspects of the chapter. We are indebted to Arnaud Costinot for insightful comments*
393 *and suggestions. Autor acknowledges support from the National Science Foundation*
394 *(CAREER award SES-0239538)*, in *Handbook of Labor Economics*, D. Card and O.
395 Ashenfelter, Editors. 2011, Elsevier. p. 1043-1171.
396 22.	Autor, D., F. Levy, and R. Murnane, *The Skill Content Of Recent Technological Change:*
397 *An Empirical Exploration.* Proceedings, 2003. **118**.
398 23.	Szymon, G., et al., *Tasks and skills in European labour markets. Background paper for*
399 *the World Bank report “Growing United: Upgrading Europe’s Convergence Machine”*.
400 2017, Instytut Badan Strukturalnych.
401 24.	Frey, C. and M. Osborne, *The Future of Employment: How Susceptible Are Jobs to*
402 *Computerisation?* Oxford Martin, 2013. **114**.
403 25.	Arntz, M., T. Gregory, and U. Zierahn, *Revisiting the Risk of Automation.* Economics

404	Letters, 2017. **159**: p. 157-160.

405	26.	Borland, J. and M. Coelli, *Are Robots Taking Our Jobs?* Australian Economic Review,

406	2017. **50**: p. 377-397.

407 27.	Autor, D. and D. Dorn, *This Job Is "Getting Old": Measuring Changes in Job*
408 *Opportunities Using Occupational Age Structure.* American Economic Review, 2009.
409 **99**: p. 45-51.
410 28.	Dorn, D., G. Hanson, and D. Autor, *Untangling Trade and Technology: Evidence from*
411 *Local Labour Markets.* Economic Journal, 2015. **125**.
412 29.	Rica, S., L. Gortazar, and P. Lewandowski, *Job Tasks and Wages in Developed Countries:*
413 *Evidence from PIAAC.* Labour Economics, 2020. **65**: p. 101845.
414 30.	Mean, M. and M.G. Mochel, *Job automation risk and the future of skills: Skills and*
415 *competency change in the US workforce*. 2023, Technical report, US Bureau of Labor
416 Statistics.
417 31.	Arntz, M., T. Gregory, and U. Zierahn, *The Risk of Automation for Jobs in OECD*
418 *Countries.* 2016.
419 32.	Albinowski, M. and P. Lewandowski, *The impact of ICT and robots on labour market*
420 *outcomes of demographic groups in Europe.* Labour Economics, 2024. **87**: p. 102481.
421 33.	Norway,	S.	*Utdanningsgapet	bare	øker*.	Available	from:
422 [https://www.ssb.no/befolkning/artikler-og-publikasjoner/utdanningsgapet-bare-](https://www.ssb.no/befolkning/artikler-og-publikasjoner/utdanningsgapet-bare-oker)
423 [oker](https://www.ssb.no/befolkning/artikler-og-publikasjoner/utdanningsgapet-bare-oker).

424

Figur_0_e.21

[Click here to access/download;Figure;Fig1.pdf](https://www2.cloud.editorialmanager.com/pone/download.aspx?id=38317236&guid=309a83d9-c895-4099-a8aa-08cf6a4af4b7&scheme=1)

<https://www2.cloud.editorialmanager.com/pone/download.aspx?id=38317236&guid=309a83d9-c895-4099-a8aa-08cf6a4af4b7&scheme=1>0.1

RTI z−score

0.0

−0.1

Women Men

−0.2

Year

2003

2004

2005

2006

2007

2008

2009

2010

2011

2012

2013

2014

2015

2016

2017

2018

###### Figure 2A

Low education

###### [Click here to access/download;Figure;Fig2A.pdf](https://www2.cloud.editorialmanager.com/pone/download.aspx?id=38318270&guid=dc5bda4f-3450-4a71-87ed-b60739b0d0e0&scheme=1)

<https://www2.cloud.editorialmanager.com/pone/download.aspx?id=38318270&guid=dc5bda4f-3450-4a71-87ed-b60739b0d0e0&scheme=1>Low own income Low father income

Unmarried Musculoskeletal

Gender

Female Male

Childless

###### Figure 2B

Low education

###### [Click here to access/download;Figure;Fig2B.pdf](https://www2.cloud.editorialmanager.com/pone/download.aspx?id=38318271&guid=e3b429d4-efbc-4e36-a000-f0d5b7080a16&scheme=1)

<https://www2.cloud.editorialmanager.com/pone/download.aspx?id=38318271&guid=e3b429d4-efbc-4e36-a000-f0d5b7080a16&scheme=1>Low own income Low father income

Unmarried Childless

Gender

Female Male

Musculoskeletal

###### Figure 3A

Low education

###### [Click here to access/download;Figure;Fig3A.pdf](https://www2.cloud.editorialmanager.com/pone/download.aspx?id=38317243&guid=ffccaac3-d1a0-4d3d-85ac-71645a8162d0&scheme=1)

<https://www2.cloud.editorialmanager.com/pone/download.aspx?id=38317243&guid=ffccaac3-d1a0-4d3d-85ac-71645a8162d0&scheme=1>Low own income

Musculoskeletal

Low father income

Childless

Female Male

Psychological

Unmarried

0.2	0.4	0.6

Proportion in 2003 and 2018

###### Figure 3B

Low education

###### [Click here to access/download;Figure;Fig3B.pdf](https://www2.cloud.editorialmanager.com/pone/download.aspx?id=38317244&guid=c2300d89-1593-45f2-b32b-18687f6049b0&scheme=1)

<https://www2.cloud.editorialmanager.com/pone/download.aspx?id=38317244&guid=c2300d89-1593-45f2-b32b-18687f6049b0&scheme=1>Low own income

Musculoskeletal

Low father income Psychological

Female Male

Childless

Unmarried

−0.20	−0.15	−0.10	−0.05	0.00

Change in RTI z−score

###### [Click here to access/download;Figure;Fig4.pdf](https://www2.cloud.editorialmanager.com/pone/download.aspx?id=38318282&guid=607da610-3369-4955-abf7-f8a8c5c599e2&scheme=1) [<https://www2.cloud.editorialmanager.com/pone/download.aspx?id=38318282&guid=607da610-3369-4955-abf7-f8a8c5c599e2&scheme=1>](https://www2.cloud.editorialmanager.com/pone/download.aspx?id=38318282&guid=607da610-3369-4955-abf7-f8a8c5c599e2&scheme=1)

Figure 4

Figure S1

1.0

0.5

0.0

−0.5

−1.0

1.0

0.5

0.0

−0.5

−1.0

1.0

0.5

0.0

−0.5

−1.0

1.0

0.5

0.0

−0.5

−1.0

RTI score coefficient

1.0

0.5

0.0

−0.5

−1.0

1.0

0.5

0.0

−0.5

−1.0

1.0

0.5

0.0

−0.5

Women

| [Click here tMo en](https://www2.cloud.editorialmanager.com/pone/download.aspx?id=38318283&guid=be4ab962-d31f-4504-a642-628f363e59fa&scheme=1)  [access/download;Figure;S1Fig](https://www2.cloud.editorialmanager.com/pone/download.aspx?id=38318283&guid=be4ab962-d31f-4504-a642-628f363e59fa&scheme=1) | | | | [.pdf](https://www2.cloud.editorialmanager.com/pone/download.aspx?id=38318283&guid=be4ab962-d31f-4504-a642-628f363e59fa&scheme=1) |
| --- | --- | --- | --- | --- |
|  |  |  |  | Childless |
|  |  |  |  |  |
|  |  |  |  |  |
|  |  |  |  |  |
|  |  |  |  |  |
|  |  |  |  |  |
|  |  |  |  |  |
|  |  |  |  |  |

|  |  |  |  | Low education |
| --- | --- | --- | --- | --- |
|  |  |  |  |  |
|  |  |  |  |  |
|  |  |  |  |  |
|  |  |  |  |  |
|  |  |  |  |  |
|  |  |  |  |  |
|  |  |  |  |  |

|  |  |  |  | Low father income |
| --- | --- | --- | --- | --- |
|  |  |  |  |  |
|  |  |  |  |  |
|  |  |  |  |  |
|  |  |  |  |  |
|  |  |  |  |  |
|  |  |  |  |  |
|  |  |  |  |  |

No Yes

|  |  |  |  | Low own income |
| --- | --- | --- | --- | --- |
|  |  |  |  |  |
|  |  |  |  |  |
|  |  |  |  |  |
|  |  |  |  |  |
|  |  |  |  |  |
|  |  |  |  |  |
|  |  |  |  |  |

|  |  |  |  |  |  |  | Musculoskeletal |
| --- | --- | --- | --- | --- | --- | --- | --- |
|  |  |  |  |  |  |  |  |
|  |  |  |  |  |  |  |  |
|  |  |  |  |  |  |  |  |
|  |  |  |  |  |  |  |  |
|  |  |  |  |  |  |  |  |

|  |  |  |  |  |  |  | Psychological |
| --- | --- | --- | --- | --- | --- | --- | --- |
|  |  |  |  |  |  |  |  |
|  |  |  |  |  |  |  |  |
|  |  |  |  |  |  |  |  |
|  |  |  |  |  |  |  |  |
|  |  |  |  |  |  |  |  |
|  |  |  |  |  |  |  |  |

−1.0

|  |  |  |  | Unmarried |
| --- | --- | --- | --- | --- |
|  |  |  |  |  |
|  |  |  |  |  |
|  |  |  |  |  |
|  |  |  |  |  |
|  |  |  |  |  |
|  |  |  |  |  |
|  |  |  |  |  |

2005	2010	2015	2005	2010	2015

Figure S2

0.525

Frey Osborne Index

[Click here to access/download;Figure;S2Fig.pdf](https://www2.cloud.editorialmanager.com/pone/download.aspx?id=38317249&guid=3f20ca4b-4561-4563-b849-818ef6607f53&scheme=1)

0.500

Women

0.475	Men

0.450

2003

2004

2005

2006

2007

2008

2009

2010

2011

2012

2013

2014

2015

2016

2017

2018

Figure S3

[Click here to access/download;Figure;S3Fig.pdf](https://www2.cloud.editorialmanager.com/pone/download.aspx?id=38317250&guid=31bc698c-2f25-43b2-a621-14992ecf8b43&scheme=1)

0.1

RTI z−score

0.0

−0.1

Women Men

−0.2

2003

2004

2005

2006

2007

2008

2009

2010

2011

2012

2013

2014

2015

2016

2017

2018

Figur0e.0S54

[Click here to access/download;Figure;S4Fig.pdf](https://www2.cloud.editorialmanager.com/pone/download.aspx?id=38317251&guid=a204f899-f525-4de0-92fb-05485becc5e6&scheme=1)

0.00

−0.05

RTI score

−0.10

Women Men

−0.15

2003

2004

2005

2006

2007

2008

2009

2010

2011

2012

2013

2014

2015

2016

2017

2018

Figure S5A

Low education

###### [here to s/download;Figure;S5AFig.pdf](https://www2.cloud.editorialmanager.com/pone/download.aspx?id=38318295&guid=9a3e314b-7074-43d0-8e9b-3a810caaa3b7&scheme=1)

Low own income Low father income

Unmarried Childless

Gender

Female Male

Musculoskeletal

[Click](https://www2.cloud.editorialmanager.com/pone/download.aspx?id=38318295&guid=9a3e314b-7074-43d0-8e9b-3a810caaa3b7&scheme=1)

[acces](https://www2.cloud.editorialmanager.com/pone/download.aspx?id=38318295&guid=9a3e314b-7074-43d0-8e9b-3a810caaa3b7&scheme=1)

###### Figure S5B

Low education

###### [Click here to access/download;Figure;S5BFig.pdf](https://www2.cloud.editorialmanager.com/pone/download.aspx?id=38318296&guid=2a0d1dbd-9f25-473a-bdd3-ae28da4922ef&scheme=1)

Low own income

Childless Low father income

Unmarried

Gender

Female Male

Musculoskeletal

###### Figure S6A

Low education

###### [Click here to access/download;Figure;S6AFig.pdf](https://www2.cloud.editorialmanager.com/pone/download.aspx?id=38318297&guid=a6ab167b-5ccd-41b4-bcf3-1b917f7b5458&scheme=1)

Low own income Low father income

Unmarried Musculoskeletal

Gender

Female Male

Childless

###### Figure S6B

Low education

###### [Click here to access/download;Figure;S6BFig.pdf](https://www2.cloud.editorialmanager.com/pone/download.aspx?id=38318298&guid=cbc2bcd5-0131-49ee-81b9-bbf84b7693bf&scheme=1)

Low own income Low father income

Unmarried Childless

Gender

Female Male

Musculoskeletal

###### [Click here to access/download;Figure;S7Fig.pdf](https://www2.cloud.editorialmanager.com/pone/download.aspx?id=38318299&guid=b2f805a2-4559-4ff1-bda0-fb38aa4c2a4c&scheme=1)

Figure 7

###### Supporting Information

[Click here to access/download](https://www2.cloud.editorialmanager.com/pone/download.aspx?id=38317234&guid=6a55f272-f75a-42f2-873d-9a9d45fff563&scheme=1)

[**Supporting Information** SupportingInformation.docx](https://www2.cloud.editorialmanager.com/pone/download.aspx?id=38317234&guid=6a55f272-f75a-42f2-873d-9a9d45fff563&scheme=1)

Revised Manuscript with Track Changes

### 1 The Distribution of Technology Induced Job Loss:

2 **Evidence from a Population-wide Study in Norway**

3

4

5

6 Bjørn-Atle Reme^1,*^, Ole Røgeberg^2^, Jonathan Wörn^1^, Bernt Bratsberg^2^, Vegard F.
7 Skirbekk^1^

8

9

10 ^1^Norwegian Institute of Public Health, Oslo, Norway
11 ^2^Frisch Center, Oslo, Norway

12

13 * Corresponding author
14 E-mail: [bjorn-atle.reme@fhi.no](mailto:bjorn-atle.reme@fhi.no)

15

16

17

18

### 19 Abstract

20 Globalization and automation are leading to skill-biased structural changes in the
21 labor market, resulting in the polarization of employment opportunities. There is increasing
22 concern that the relative decline of occupations characterized by routine cognitive and
23 physical tasks is contributing to growing earnings- and gender inequality. Using
24 comprehensive individual-level data from Norway, our analysis reveals significant and
25 growing gender disparities in the routine intensity of occupations. Additionally, we show
26 that the gender disparities are particularly strong among individuals with low socioeconomic
27 status, and that the main contributor to the growing gender disparities is the increasing
28 gender difference in educational attainment.

### 29 Introduction

30 In recent decades, skill-biased technological change has allowed firms to automate
31 and offshore routine-intensive tasks, leading to an erosion of low skilled and mid-level jobs
32 and increasing returns to education [1-4]. These trends are expected to continue, and
33 according to the OECD, approximately 14% of jobs are at risk of automation [5]. ~~While the~~
34 ~~disappearance of routine-intensive occupations may not necessarily result in an increase in~~
35 ~~long-term unemployment (Gregory et al., 2021), it does result in a shift in the labor market~~
36 ~~structure.~~While the disappearance of routine-intensive occupations may not necessarily
37 result in an increase in long-term unemployment [6], it does result in a shift in the labor
38 market structure [7, 8]. In fact, a recent OECD policy-brief found that countries with higher
39 investments in robotics experienced *higher* employment growth ~~[6]~~[9], but also heightened
40 job instability among low-educated workers, typically concentrated within occupations with
41 a high risk of automation. This raises the question of whether a continuation of these trends
42 will serve to amplify or dampen pre-existing inequalities [~~7, 8~~10, 11].
43 Several studies find that less-educated men is a group that has experienced stagnation
44 or declines in both wage level and labor market participation ~~[9].~~[12, 13]. This development
45 has coincided with a decline in marriage rates, fertility, and health outcomes [~~10-12~~13-15].
46 While the reasons behind these developments are largely unknown, it has stirred debates
47 among policy makers and academics as to the role of technological development. ~~The~~
48 ~~purpose of this study was to examine this relationship more explicitly. Specifically, the study~~
49 ~~aimed to investigate the sex-specific relationship between a widely used indicator of~~
50 ~~structural risk at the occupation level and indicators of socioeconomic status and welfare~~
51 ~~using Norwegian administrative registers. Specifically, we aimed to (i) examine and explain~~
52 ~~the development in sex~~
53 Norway is a small, open economy with a highly educated workforce, extensive social
54 safety net, and a compressed wage structure with centralized wage bargaining and strong
55 labor unions. These factors have been argued to promote the adoption of technological
56 innovations by industry [16]. Over recent decades, Norway has experienced both an increase
57 in employment within high-skill occupations and a rise in automation through the use of
58 industrial robots, reflecting broader trends observed in other developed economies [17, 18].
59 Recent evidence from Norway suggests that while automation has negative impacts on
60 employment and wages, these effects are, to some extent, mitigated by labor unions [19].
61 While previous studies primarily have focused on the negative impacts of automation on
62 employment or inequality, less is known regarding the distribution of this risk across the
63 population and to what extent we should expect it to dampen or amplify pre-existing
64 socioeconomic differences. In particular, evidence on the development over time within

**Formatert:** Tabulatorstopp: 8,75 cm, Left

65 subgroups and its association with other known risk factors of social marginalization, is
66 scarce. We hypothesize that men with lower education, who are more likely to be in routine-
67 based occupations requiring fewer social skills, are at an increasingly higher risk of
68 automation. Such a trend could exacerbate existing social inequalities [20].
69 The study aimed to investigate how a widely used indicator of occupational
70 automation risk is associated and aligned at the individual level with measures of
71 socioeconomic status in a register based study of the full population of Norwegian 45-year-
72 olds, and how this differs for men and women. Our specific objectives were to (i) examine
73 and explain the development in gender-specific risk across birth cohorts, and (ii) to estimate
74 the association with other risk factors of social marginalization, including educational
75 attainment, income, family background and health measures.

### 76 Methods

#### 77 Data and analytical sample

78 ~~We used administrative register data linked and deidentified by Statistics Norway,~~
79 ~~covering~~We used linked population-wide administrative register data, covering information
80 from several national registries. The registers were linked by Statistics Norway and then
81 deidentified before researchers were allowed access. The acquisition of the data was part of
82 the data structure at the Center for Fertility at the Norwegian Institute of Public Health, where
83 data sets from the different registers were linked and stored at a secure server hosted by the
84 Services for sensitive data (TSD), University of Oslo.
85 The information included in the database was demographic information and family
86 status (national population register), labor market history (employer and employee register),
87 educational attainment (national educational database), and physician visits (Norway
88 Control and Payment of Health Reimbursements database). The data were accessed by the
89 researchers for research purposes on March 1st, 2020.

**formaterte:** Ikke Hevet/ Senket

90 Our sample population was all individuals born 1958-1973 who were employed in
91 the year they turned 45 (N = 900,559) and living in Norway. Since there are considerable
92 differences in early-career trajectories across education levels, we measured the structural
93 risk of automation of the occupation held mid-career, at age 45. Our study period covers
94 2003-2018.
95 ~~To measure structural risk of automation at the occupation level, we use the routine~~
96 ~~task intensity (RTI) index suggested in Acemoglu and Autor (2011), and as operationalized~~
97 ~~in Lewandowski et al. (2017) (see Supplementary material for details). The RTI score is~~
98 ~~calculated from the occupational task descriptions in the O*NET database and reflects the~~
99 ~~extent to occupations consist of routine-based tasks and require interpersonal interaction.~~
100 ~~The RTI-measure is built on the assumption that jobs with high routine intensity and/or low~~
101 ~~levels of interpersonal interaction are more prone to automation. To ease interpretation, we~~
102 ~~standardized the RTI score distribution in the sample to be mean zero with a standard~~
103 ~~deviation of 1 (RTI z-score).~~
104 ~~The RTI is one of two commonly used measures used to assess the risk of automation,~~
105 ~~with the other being the Frey-Osborne index (FOI) [13, 14]. The main difference between~~
106 ~~these measures is that the RTI is task-based – it assesses to what extent a job consists of tasks~~
107 ~~that could be automated – while the FOI is based on expert assessment of occupations. The~~
108 ~~measures are correlated, but RTI has a stronger theoretical foundation, allows for~~
109 ~~occupational scores to change with their task content [15], has done comparatively better in~~
110 ~~predictive tests [16], and has been used in an extensive literature on skill biased technological~~
111 ~~change [2, 13, 17-19] (see [20] and [21] for a description and discussion of the difference~~
112 ~~between these measures, and other alternative measures used in the literature).~~
113 To measure structural risk of automation at the occupation level, we use the routine
114 task intensity (RTI) index suggested in Acemoglu and Autor (2011)[21]. This approach to
115 measuring routine intensity has been applied extensively in the labor economics literature
116 on the impacts of skill based technological change, starting with the seminal contribution of
117 Autor et al. (2003)[22]. The measure is a theoretically informed indicator of structural
118 economic risk, classifying occupations based on the tasks performed in it. In particular, the
119 index captures the extent to which an occupation is characterized by routine tasks in the
120 cognitive, manual or interpersonal domain. This reflects an economic theory of automation
121 predicting that routine tasks are more easily automated, an assumption that has received
122 extensive empirical support in work employing the RTI index [22]. Measurement of the
123 content of job tasks were retrieved from the O*NET database. This database consists of a
124 highly detailed description of tasks performed in an occupation. From the large set of
125 measurements in O*NET, Acemoglu and Autor (2011) suggests a subset of items which
126 describe these dimensions. To summarize these items to a one-dimensional RTI measure for
127 each occupation, we used the method suggested by Lewandowski et al. (2017) (see
128 Supplementary material for details) [23]. Last, to ease interpretation, we standardized the
129 RTI score distribution in the sample to be mean zero with a standard deviation of 1 (RTI z-
130 score).
131 The RTI is one of two commonly used measures used to assess the risk of automation,
132 with the other being the Frey-Osborne index (FOI) [22, 24]. The main difference between
133 these measures is that the RTI is task-based – it assesses to what extent a job consists of tasks
134 that could be automated – while the FOI is based on expert assessment of occupations. The
135 measures are correlated, but RTI has a stronger theoretical foundation, allows for
136 occupational scores to change with their task content [25], has done comparatively better in
137 predictive tests [26], and has been used in an extensive literature on skill biased technological
138 change [2, 22, 27-29] (see [30] and [31] for a description and discussion of the difference
139 between these measures, and other alternative measures used in the literature).
140 The main analysis in this paper is based on the 2003 version of the RTI – the first
141 year of our study period. However, in the Supplementary material we also present all main
142 results using both the FOI and the RTI based on 2019 O*Net-data to assess robustness of
143 our conclusions against changes in task content over time. S1 Table shows that all these
144 measures of structural change are highly correlated, and our main conclusions are therefore
145 independent of the selected measure.
146 Other measures used as covariates for stratification in the analysis were the following:
147 Education: The highest level of completed education, available from the National Education
148 Database. There are 9 levels within the ISCED classification, where level 9 is “unknown”.
149 In the analysis we either use 4 categories: primary (1-2), secondary (3-5), 1–4-year university
150 education (6) and master and higher university education (7-8), or dichotomize by whether
151 or not the individual had university level education or not, i.e. 1-5 vs 6-8, referred to as “low”
152 and “high” education, respectively.
153 Own income: Own income was retrieved from the income tax register. We calculated
154 birthyear- and gender-stratified income quintiles. In the main part of the analysis, we use a
155 binary variable indicating whether the individual belonged to the lowest income quintile.
156 ~~Paternal~~Father income: ~~Father~~Father’s income was retrieved from the income tax register.
157 We calculated the father’s income quintile at age 40, stratified on his birth cohort. In the
158 main part of the analysis, we use a binary variable indicating whether the father belonged to
159 the lowest income quintile.
160 Marital status: Marital status was retrieved from the official marriage register. This contains
161 complete records of marriages in Norway since 1974. For the analysis we used a binary
162 indicator for whether the individual was registered as married at age 45.
163 Childlessness: Using the population register we created a binary indicator for whether the
164 individual was childless at age 45.
165 Musculoskeletal/psychological problems: We used the register of primary care utilization
166 (KUHR) to create a binary indicator for whether the individual had visited primary health
167 care (general practitioner) for musculoskeletal (L-chapter symptoms or diagnoses) and/or
168 psychological (P-chapter symptoms or diagnoses) problems during the year when being 45-
169 years old. These two chapters were chosen, as they are the most common reasons for both
170 health problems and sick leave among 45-year-olds in Norway.
171 Table 1 provides summary statistics across covariates, excluding covariates based on
172 creating groups based on the underlying distribution (quintiles for own income and parental
173 income).
174 Table 1. Summary statistic for covariates

| **Covariate** | **Proportion** |
| --- | --- |
| Female | 49% |
| Childless | 15% |
| Married | 57% |
| ~~Elementary~~Primary education | 25% |
| Secondary education | 36% |
| ~~University bachelor~~Low university education | 29% |
| ~~University Master~~High university (Master’s | 10% |
| Visit to doctor for psychological problem | 9% |
| Visit to doctor for musculoskeletal problem | 22% |

Number of observations (N)	900,559

| 175 |  |
| --- | --- |
| 176 | **Empirical strategy and statistical analysis** |
| 177 | The aim was to examine the relationship between structural risk of automation at the |
| 178 | occupation level and indicators of socioeconomic status. All individuals in our sample were |
| 179 | assigned an RTI score based on the occupation they had at age 45. Our aim was then to |
| 180 | assess how this occupational risk score was associated with other measures of socioeconomic |
| 181 | risk factors. To assess the extent of structural shifts in the labor market throughout our |
| 182 | sample period we estimated the year- and gender-specific average RTI z-score for 45-year- |
| 183 | olds. |
| 184 | We then examined how structural risk of automation (RTI z-score) was associated |
| 185 | with our measures of socioeconomic status and health: Using simple and multivariate |
| 186 | regression models separately for each ~~sex~~gender, we assess the extent to which RTI score is |
| 187 | predicted by the other measures, with birth cohort dummies used to control for potential time |
| 188 | trends. |
| 189 | Next, we performed an attribution analysis where we estimated how much of the |
| 190 | increase in automation risk gender gap can be explained by the various covariates included |
| 191 | in the regression models. |
| 192 | Finally, to examine the overall variation in occupational risk scores in most current |
| 193 | population of 45-year-olds in our data - birth cohort 1973 - we predicted the RTI z-score of |
| 194 | men and women for each observed combination of covariate values and plotted the ordered |
| 195 | estimates in a bubble scatter plot where the bubble size reflects the size of the covariate |
| 196 | group. To examine how these patterns have evolved over time, we also reproduced the plot |
| 197 | for the first birth cohort in our data (born in 1958). |

#### 198 Ethical considerations

199 The ethical approval for this study was given by The Ethics Committee of South-East
200 Norway (#2018/434). Based on Section 35 of the Health Research Act, participant consent
201 was waived by the Committee. Data from the different registers were linked by certified
202 researchers who only had access to encrypted personal ID-variable. Extensive measures
203 were taken to maintain the security and confidential handling of the research data.
204 Throughout the process of analyzing data and presenting findings, we are committed to
205 preventing stigmatization and to upholding ethical principles of honesty and transparency in
206 reporting our findings. To achieve this, we used neutral language and avoided portraying
207 vulnerable groups in ways that might be perceived as burdensome. Additionally, we
208 refrained from reporting on groups small enough to risk identifying individuals.

### 209 Results

#### 210 Gender specific time trends in routine intensity

211 Our data covers the 15-year period 2003 to 2018. We first examined the development
212 in routine intensity across this period, separately for each ~~sex~~gender (Figure 1). In the
213 supplementary materials we also present the development in RTI score for each of the
214 covariates used in the analysis (S1 Fig).

215

**formaterte:** Skrift: 12 pkt

216 Fig 1. The yearly average ~~RTI score~~routine intensity index among 45-year-olds by
217 **gender.** The figure shows the yearly average RTI z-score, stratified by year, for 45-year-
218 olds~~, i.e.,~~ (birth cohorts 1958 to 1973) by gender. The 95% confidence intervals are indicated
219 by the error bars. See S2 Table in the Supplementary Material for a corresponding table.

220 While there already was a substantial gender difference in standardized routine

221	intensity in 2003 - the score was 0.13 [95% CI, 0.12-0.14] for men and -0.13 [95% CI, (-

222 0.14)-(-0.12)] for women - the difference gradually increased up until 2018, to 0.18 [95%
223 CI, 0.17-0.19] for men and -0.20 [95% CI, (-0.21)-(-0.19)] for women. In the Supplementary
224 material we also present the average risk score using the Frey-Osbourne index (S2 Fig), RTI
225 2019 z-score (S3 Fig), and not z-standardized RTI scores (S4 Fig). It should be noted that
226 when presenting time trends with the FOI or raw RTI-score, the time trend is downward
227 sloping, indicating that the overall risk of automation is falling during the study period.
228 However, the decrease is smaller for men, causing an increasing gender gap.

#### 229 The association between structural risk and socioeconomic status

230 In bivariate analyses, RTI z-score was most strongly associated with low education
231 (education below university level) with coefficients of 1.11 (p < 0.001) for women and 0.82
232 for men (p < 0.001) (Figure 2A). Regarding gender, the association between RTI z-score and
233 socioeconomic risk factors were higher among men for almost all socioeconomic indicators
234 assessed. The exceptions were females who were childless or with a psychological diagnosis
235 from their physician. See the Supplementary material for models using the Frey-Osbourne
236 index (S5 Fig) and RTI 2019 z-score (S6 Fig).

237

**Formatert:** Innrykk: Første linje: 0 cm, Mellomrom Etter: 9,55 pkt.

**formaterte:** Skrift: Fet

238 **~~Fig~~Figure 2. ~~RTI~~Routine intensity risk factors ~~across~~by gender.** The figure ~~shows the~~

**formaterte:** Skrift: Ikke Fet

239 ~~coefficient results (with 95% CIs)~~displays regression coefficients from (A) bivariate ~~(panel~~
240 ~~A)~~ and (B) multivariate ~~(panel B)~~ regression models, with the RTI z-score as the outcome
241 variable ~~among~~for all 45-year-olds ~~in the period~~between 2003- and 2018, separately ~~for~~
242 ~~each~~by gender. ~~Corresponding~~The 95% confidence intervals are indicated by the error bars.

243 Detailed regression tables are ~~included~~provided in the Supplementary ~~material~~Material (S2
244 Table).

**formaterte:** Skrift: Ikke Fet

#### 245 Explaining the increasing gender gap – attribution analysis

246 The gender differences in regression coefficients shown in Figure 2 could explain a
247 gender gap in the RTI. However, to ~~explain~~understand why ~~the gender~~this gap ~~is~~
248 ~~increasing~~has increased over time, we also need ~~information on~~ to consider gender-specific
249 changes in the prevalence of ~~these~~relevant risk factors ~~over time~~. To ~~shed~~explore this further
250 ~~light on this issue~~, we ~~therefore~~ conduct an attribution analysis ~~where we estimate~~to assess
251 how the ~~sex~~gender-specific development of each risk factor contributes to the overall change
252 in the ~~share of the sample exposed to a given risk factor.~~gap. This analysis ~~consists of~~was
253 carried out in two ~~parts: first~~steps. First, the gender-specific change in ~~proportion of the~~
254 ~~sample that are exposed to a given risk factor~~ prevalence over the period 2003 to 2018 was
255 calculated (Figure 3A). Second, ~~this change is then~~to estimate the contribution, we
256 multiplied ~~with~~these changes by the regression coefficients ~~estimated in~~from the
257 multivariate model (~~cf.~~see Figure 2B) to ~~provide an~~ estimate the relative impact of each risk
258 factor to the ~~relative importance of the different risk factors for the development of~~ evolving
259 gender-specific risk of automation ~~over time~~. These results are shown in Figure 3B.
260 ~~Fig~~
261 **Figure 3. Attribution analysis~~.~~** ~~Panel A shows the share~~ **of routine intensity risk factors**

**Formatert:** Mellomrom Etter: 6 pkt.

**formaterte:** Skrift: Fet

262 **among 45-year-olds in 2003 and 2018** ~~that were~~**, by gender.** (A) Share of 45-year-olds in

**formaterte:** Skrift: Fet

263 2003 and 2018 exposed to ~~the different~~various risk factors. ~~Panel~~ (B ~~shows the~~) Estimated
264 impact of ~~this change~~changes in exposure, calculated by multiplying the change in share (~~as~~
265 ~~shown in panel~~from Panel A) ~~with~~by the corresponding regression coefficient ~~of the given~~for
266 each characteristic ~~from~~, based on a multivariate regression of the RTI z-score on all risk
267 factors (~~multivariate regression –~~ see Figure 2B).
268 The largest change in risk factor exposure was related to an increase in higher
269 education (and correspondingly a decline in the share with low education; Figure 3A),
270 especially among women (from 33 to 58 percent). There was also a substantial increase in
271 the share of the sample that were unmarried, both among men (from 37 to 49 percent) and
272 women (35 to 47 percent). Regarding the impact of the change in exposure on RTI z-score,
273 i.e., when combining the strength of association (Figure 2B) with the change in exposure
274 (Figure 3A), the strongest impact was found for education with an estimated impact on the
275 RTI z-score of -0.117 (95% CI (-0.116)-(-0.118)) for men and -0.197 (95% CI (-0.195)-(-
276 0.198)) for women.

#### 277 Distribution of structural risk across population groups

278 The estimated RTI z-scores from a multivariate model applied to the 2018 cohort of
279 45 years old individuals (birth cohort 1973) showed substantial variation across
280 combinations of risk factors (Figure 3). While the predicted RTI z-scores were -0.75 [95%
281 CI (-0.78)-(-0.72)] and -0.80 [95% CI (-0.83)-(-0.77) ] among highly educated married men
282 and women with children and from high SES families, the corresponding RTI z-scores were
283 0.82 [95% CI 0.69-0.95] and 0.37 [95% CI 0.17-0.58] among less educated unmarried men
284 and women without children from low SES families (see S3 Table and S4 Table for
285 corresponding tables). To examine how this distribution has developed over time, we also
286 estimated this model for the 2003 cohort (1958 birth cohort). The comparison across cohorts
287 reveals that while the gender difference in automation risk declined among highly educated
288 individuals, it has remained substantial among those of lower education. Hence, considering
289 the increasing share of women taking higher education, it supports our finding that the
290 increasing gender gap is due to a large shift in the share of women with university education
291 (S7 Fig).
292 ~~Fig~~
293 **Figure 4. Predicted RTI z-scores from multivariate regression by gender.** The figure

**formaterte:** Skrift: Ikke Fet

294 ~~shows the~~presents predicted RTI z-scores ~~from a model~~based on gender-specific models
295 estimated ~~on~~for the 1973 birth cohort (i.e., those aged 45 ~~years old~~ in 2018~~) explaining RTI z-~~
296 ~~score with four~~ ). The explanatory variables were indicators for educational ~~levels, a dummy~~
297 ~~for childless, a dummy for~~ level (Primary educ = primary education; Secondary educ =
298 secondary education; Low uni = lower university (Bachelor’s degree); High uni = higher
299 university (Master’s degree or higher)), indicators for childlessness (Childless), marital status
300 (Married), and whether ~~married and a dummy for whether~~ the father’s ~~income~~ average
301 income rank between ages 40 and 50 was within the lowest quintile ~~of~~among men of
302 ~~similar~~the same age. group (Low parental income). See Supplementary Material (S3 Table
303 and S4 Table) for ~~corresponding~~detailed tables, and the average RTI z-scores for each group.

### 304 Discussion

305 Our study has four main findings. First, the risk of automation was higher among
306 men than women. This difference increased from 2003 to 2018. Second, the risk of
307 automation was higher among individuals with low socioeconomic status and less social
308 support in the form of a partner and children. Third, the main reason for the increasing gender
309 gap was the growing gender differences in educational attainment. In summary, our study
310 suggests that automation may exacerbate existing social inequalities and create a more
311 unstable job market situation for men that may already struggle financially and socially. And,
312 if disparities in economic and social opportunity are allowed to grow without mitigating
313 policies, they have the potential to create social unrest and political polarization. At the same
314 time, the nature of technological development changes rapidly, as evidenced by the
315 introduction of large language models and multimodal models in recent years. These new
316 developments may have further increased the scope of automation, potentially increasing the
317 scope of routine tasks to also cover language-based tasks such as report writing and analysis.
318 Gender disparities in the risk of automation have also been found in other studies
319 covering several European countries ~~[21].~~[31]. Interestingly, whether men or women are
320 found to be at the higher risk of automation varies across countries, with the Nordic countries
321 having a higher risk among men. There could be several reasons for such differences across
322 countries, for example related to the Nordic countries having strong gender equality norms,
323 high growth in the education level among women, and lower levels of female part-time work.
324 Moreover, our study covers prime-aged individuals, and the risk likely varies substantially
325 across age. The importance of age is documented in a recent study assessing the causal
326 impact of robot- and information technology across several European countries. While the
327 impact of these technologies on employment were more negative among older women,
328 compared to older men, the impact in prime age (age 30-49) was considerably more negative
329 for men than for women ~~[22].~~[32].
330 We have found that the growing gender gap in the risk of automation can be
331 attributed, in large part, to the increasing levels of education among women. It is interesting
332 to note that this gap in education continues to widen, and Statistics Norway reported in 2019
333 that in many municipalities the share of highly educated women is more than twice that of
334 men ~~[23].~~[33].
335 As these developments are likely to continue, it is important for policy makers to
336 understand their implications and carefully consider the best ways to address and mitigate
337 the negative impacts.

#### 338 Strengths and weaknesses

339 The main strength of our study is that it covers the full population of employed 45-
340 year-old individuals in Norway over a 15-year period across several important characteristics
341 related to health, financial position, education, and family formation. Hence, in contrast to
342 most other studies on this topic which uses survey data, our study does not suffer from
343 selection bias. At the same time, our study has several weaknesses. First, the study has
344 limited generalizability due to its focus on Norway, a country with unique characteristics
345 related to gender roles, institutional environments, and industry structure. For example,
346 Norway has an extensive welfare system that compensates 62.4 percent of the income in the
347 case of job loss. Hence, the external validity of the results is limited, and likely most relevant
348 for Northern European countries with similar institutions and social norms. At the same time,
349 similar gender-based occupational patterns are likely to exist in diverse geographic contexts.
350 Consequently, the widening gender gap in automation risk and its association with social
351 risk factors are likely present in countries also far from Norway. Second, the scope of the
352 study is limited to what can be quantitatively measured in administrative registers and may
353 not capture important experiential aspects related to technological development that could
354 be measured with qualitative methods.

### 355 Conclusion

356 The gender difference in the risk of automation has been increasing, with a particular
357 high risk among lower educated men with fewer family ties. This potentially has significant
358 and far-reaching negative impacts on individuals and society as it could exacerbate the
359 concentration of economic, social and health exclusion in the coming decades. Given the
360 increasing disparities identified in this study, policy makers should monitor the development
361 in job market opportunities for various subgroups of the working-age population, and
362 evaluate programs aimed at supporting individuals that are particularly negatively affected.
363 For example, given the heightened risk faced by individuals with lower levels of education,
364 policymakers should consider implementing targeted upskilling programs that equip these
365 workers—especially younger individuals—with skills that are in high demand and less likely
366 to be at risk of automation. Last, provided the recent rapid developments in generative AI,
367 future research should also explore how the risk distribution is evolving over time across
368 age-groups, industries and social risk factors.

369

### 370 Acknowledgements

371 Authors' contributions
372 O.R. and V.S. conceptualized the idea. B-A.R. and O.R. wrote the main manuscript. B-A.R.
373 were responsible for the statistical analysis. All authors contributed to designing the research
374 and the empirical strategy. All authors reviewed the manuscript.

375

376 Availability of data and materials
377 The data used in this study encompasses educational outcomes, income, employment records,
378 health records and demographic information for entire cohorts of the Norwegian population.
379 These data were made available on loan for research purposes. Other researchers may apply
380 for access to the same sources - see “helsedata.no/en” and “https://[www.ssb.no/en/data-til-](http://www.ssb.no/en/data-til-)
381 forskning/utlan-av-data-til-forskere”.

### 382 References

383 1.	Autor, D.H. and D. Dorn, *The Growth of Low-Skill Service Jobs and the Polarization of*

**formaterte:** Engelsk (USA)

384 *the US Labor Market.* American Economic Review, 2013. **103**(5): p. 1553-97.
385 2.	Goos, M., et al., *Job Polarization in Europe.* American Economic Review, 2009. **99**: p.

386	58-63.

387 3.	Goos, M., A. Manning, and A. Salomons, *Explaining Job Polarization: Routine-Biased*
388 *Technological Change and Offshoring.* The American Economic Review, 2014. **104**.
389 4.	Graetz, G. and G. Michaels, *Robots at Work.* The Review of Economics and Statistics,

390	2018. **100**(5): p. 753-768.

391 5.	Nedelkoska, L. and G. Quintini, *Automation, skills use and training.* 2018.
392 ~~6~~6.	Genz, S., et al., *How Do Workers Adjust When Firms Adopt New Technologies?* 2021.
393 7.	Acemoglu, D., C. Lelarge, and P. Restrepo, *Competing with Robots: Firm-Level Evidence*
394 *from France.* AEA Papers and Proceedings, 2020. **110**: p. 383–88.
395 8.	Acemoglu, D. and P. Restrepo, *Robots and Jobs: Evidence from US Labor Markets.*
396 Journal of Political Economy, 2020. **128**(6): p. 2188-2244.
397 9.	Gregory, T., A. Salomons, and U. Zierahn, *Racing With or Against the Machine?*

**formaterte:** Engelsk (USA)

398 *Evidence on the Role of Trade in Europe.* Journal of the European Economic
399 Association, 2022. **20**(2): p. 869-906.
400 ~~7~~10.	Bessen, J., *Automation and jobs: when technology boosts employment*.* Economic

**formaterte:** Engelsk (USA)

401	Policy, 2019. **34**(100): p. 589-626.

**formaterte:** Engelsk (USA)

402 ~~8~~11.	Schmidpeter, B. and R. Winter-Ebmer, *Automation, unemployment, and the role of*
403 *labor market training.* European Economic Review, 2021. **137**: p. 103808.

**formaterte:** Engelsk (USA)

404 ~~9~~12.	Binder, A. and J. Bound, *The Declining Labor Market Prospects of Less-Educated Men.*
405 Journal of Economic Perspectives, 2019. **33**: p. 163-190.

**formaterte:** Engelsk (USA)

406 ~~10~~13. Bratsberg, B., A. Kotsadam, and S. Walther, *Male Fertility: Facts, Distribution and*
407 *Drivers of Inequality.* SSRN Electronic Journal, 2021.

**formaterte:** Engelsk (USA)

408 ~~11~~14. Lundberg, S., R. Pollak, and J. Stearns, *Family Inequality: Diverging Patterns in*
409 *Marriage, Cohabitation, and Childbearing †.* Journal of Economic Perspectives, 2016.
410 **30**: p. 79-102.

**formaterte:** Engelsk (USA)

411 ~~12~~15. Case, A. and A. Deaton, *Rising Morbidity and Mortality in Midlife Among White Non-*
412 *Hispanic Americans in the 21st Century.* Proceedings of the National Academy of
413 Sciences, 2015. **112**: p. 201518393.
414 ~~13~~16. Barth, E., K.O. Moene, and F. Willumsen, *The Scandinavian model—An interpretation.*
415 Journal of Public Economics, 2014. **117**: p. 60-72.
416 17.	Jurkat, A., R. Klump, and F. Schneider, *Tracking the Rise of Robots: A Survey of the IFR*
417 *Database and its Applications*. 2021.
418 18.	Alasoini, T., et al., *Digital Transformations of Traditional Work in the Nordic Countries*
419 *- Edited by Bertil Rolandsson*. 2020.
420 19.	Umblijs, J., P. Schone, and H. Finseraas, *Automation and worker organisation.* Applied
421 Economics Letters, 2024: p. 1-5.
422 20.	Moll, B., L. Rachel, and P. Restrepo, *Uneven Growth: Automation's Impact on Income*
423 *and Wealth Inequality.* Econometrica, 2022. **90**(6): p. 2645-2683.
424 21.	Acemoglu, D. and D. Autor, *Chapter 12 - Skills, Tasks and Technologies: Implications*
425 *for Employment and Earnings**We thank Amir Kermani for outstanding research*
426 *assistance and Melanie Wasserman for persistent, meticulous and ingenious work on*

| 427 *all aspects of the chapter. We are indebted to Arnaud Costinot for insightful comments* 428 *and suggestions. Autor acknowledges support from the National Science Foundation* 429 *(CAREER award SES-0239538)*, in *Handbook of Labor Economics*, D. Card and O. 430 Ashenfelter, Editors. 2011, Elsevier. p. 1043-1171. 431 22. Autor, D., F. Levy, and R. Murnane, *The Skill Content Of Recent Technological Change:* 432 *An Empirical Exploration.* Proceedings, 2003. **118**. 433 ~~14~~23. Szymon, G., et al., *Tasks and skills in European labour markets. Background paper for* 434 *the World Bank report “Growing United: Upgrading Europe’s Convergence Machine”*. 435 2017, Instytut Badan Strukturalnych. | | |  | **formaterte:** Engelsk (USA) |
| --- | --- | --- | --- | --- |
| 436 | 24. | Frey, C. and M. Osborne, *The Future of Employment: How Susceptible Are Jobs to* |  | **formaterte:** Engelsk (USA) |
| 437 |  | *Computerisation?* Oxford Martin, 2013. **114**. |  |  |
| 438 | ~~15~~25. | Arntz, M., T. Gregory, and U. Zierahn, *Revisiting the Risk of Automation.* Economics |  | **formaterte:** Engelsk (USA) |
| 439 |  | Letters, 2017. **159**: p. 157-160. |  |  |
| 440 | ~~16~~26. | Borland, J. and M. Coelli, *Are Robots Taking Our Jobs?* Australian Economic Review, |  | **formaterte:** Engelsk (USA) |
| 441 |  | 2017. **50**: p. 377-397. |  |  |
| 442 | ~~17~~27. | Autor, D. and D. Dorn, *This Job Is "Getting Old": Measuring Changes in Job* |  | **formaterte:** Engelsk (USA) |
| 443 |  | *Opportunities Using Occupational Age Structure.* American Economic Review, 2009. |  |  |
| 444 |  | **99**: p. 45-51. |  |  |
| 445 | ~~18~~28. | Dorn, D., G. Hanson, and D. Autor, *Untangling Trade and Technology: Evidence from* |  | **formaterte:** Engelsk (USA) |
| 446 |  | *Local Labour Markets.* Economic Journal, 2015. **125**. |  |  |
| 447 | ~~19~~29. | Rica, S., L. Gortazar, and P. Lewandowski, *Job Tasks and Wages in Developed Countries:* |  | **formaterte:** Engelsk (USA) |
| 448 |  | *Evidence from PIAAC.* Labour Economics, 2020. **65**: p. 101845. |  |  |
| 449 | ~~20~~30. | Mean, M. and M.G. Mochel, *Job automation risk and the future of skills: Skills and* |  | **formaterte:** Engelsk (USA) |
| 450 |  | *competency change in the US workforce*. 2023, Technical report, US Bureau of Labor |  |  |
| 451 |  | Statistics. |  |  |
| 452 | ~~21~~31. | Arntz, M., T. Gregory, and U. Zierahn, *The Risk of Automation for Jobs in OECD* |  | **formaterte:** Engelsk (USA) |
| 453 |  | *Countries.* 2016. |  |  |
| 454 | ~~22~~32. | Albinowski, M. and P. Lewandowski, *The impact of ICT and robots on labour market* |  | **formaterte:** Engelsk (USA) |
| 455  456 | ~~23~~33. | *outcomes of demographic groups in Europe.* Labour Economics, 2024. **87**: p. 102481. Norway, S. *Utdanningsgapet bare øker*. Available from: |  |  |

457	[https://www.ssb.no/befolkning/artikler-og-publikasjoner/utdanningsgapet-bare-](https://www.ssb.no/befolkning/artikler-og-publikasjoner/utdanningsgapet-bare-oker)

458	[oker.](https://www.ssb.no/befolkning/artikler-og-publikasjoner/utdanningsgapet-bare-oker)

459

Response to Reviewers

##### PONE-D-23-25076R1 Response letter General comment to both reviewers

We sincerely thank the reviewers for taking their time to assess our study and for offering insightful comments and constructive feedback. Below, we address the comments and explain how we have taken them into account in the manuscripts. In cases where we reference our revised manuscript and have made additions to the existing text, the changes are highlighted in yellow.

**Note:** Page numbers and line numbers referred to in this letter are from the tracked changes version of the manuscript (page number in the middle of the bottom of each page).

##### Reviewer #3:

1. *Introduction*
   - *Clarification of research background: While the introduction already mentions the impact of technological changes on the labor market, further refinement of this background, particularly in the context of Norway, would be beneficial. For instance, specific Norwegian policies or trends in automation and globalization could be highlighted.*

##### Response:

We have added some more context, both related to the Norwegian economy and trends in automation, on page 3, line 53:

“Norway is a small, open economy with a highly educated workforce, extensive social safety net, and a compressed wage structure with centralized wage bargaining and strong labor unions. These factors have been argued to promote the adoption of technological innovations by industry [16]. Over recent decades, Norway has experienced both an increase in employment within high-skill occupations and a rise in automation through the use of industrial robots, reflecting broader trends observed in other developed economies [17, 18].”

*- Precision in stating research questions: In the introduction, it would be clearer to delineate the research questions, such as specifying which gender and socioeconomic disparities are to be investigated and how these disparities evolve over time.*

##### Response:

Based on this comment we have increased the precision of our research questions, on page 4, line 69 (changes highlighted):

“The study aimed to investigate how a widely used indicator of occupational automation risk is associated and aligned at the individual level with measures of socioeconomic status in a register based study of the full population of Norwegian 45-year-olds, and how this differs for men and women. Our specific objectives were to (i) examine and explain the development in sex-specific risk across birth cohorts, and (ii) to estimate the association with other risk factors of social marginalization, including educational attainment, income, family background and health measures.”

2. *Literature Review*
   - *Exhaustiveness of literature citations: Although the paper references some relevant literature, incorporating more recent and authoritative sources to support the arguments, especially studies related to Norway or other European countries, would strengthen the review.*

##### Response:

We have added several relevant, recent and authoritative sources to support our argument.

The following was added in the introduction, [7] and [8] are highly authoritative authors within this field, [19] is a recent study from Norway, and [20] is a paper building a theoretical argument for the distributional effects of automation in a very prestigious academic journal.

7. Acemoglu, D., C. Lelarge, and P. Restrepo, Competing with Robots: Firm-Level Evidence from France. AEA Papers and Proceedings, 2020. 110: p. 383–88.
8. Acemoglu, D. and P. Restrepo, Robots and Jobs: Evidence from US Labor Markets. Journal of Political Economy, 2020. 128(6): p. 2188-2244.
19. Umblijs, J., P. Schone, and H. Finseraas, Automation and worker organisation. Applied Economics Letters, 2024: p. 1-5.
20. Moll, B., L. Rachel, and P. Restrepo, Uneven Growth: Automation's Impact on Income and Wealth Inequality. Econometrica, 2022. 90(6): p. 2645-2683.

*- Critical analysis of existing studies: When reviewing existing literature, a critical analysis of previous research should be conducted, identifying their limitations or research gaps, thereby clearly articulating the contribution of this study.*

##### Response:

We have added a clearer statement of our contribution in relation to the literature, on page 3, line 61:

“While previous studies primarily have focused on the negative impacts of automation on employment or inequality, less is known regarding the *distribution* of this risk across the population and to what extent we should expect it to dampen or amplify pre-existing socioeconomic differences. In particular, evidence on the development over time within subgroups and its association with other known risk factors of social marginalization, is scarce.”

3. *Methods*
   - *Detailed description of data: When describing the data sources and analysis sample, provide a more detailed account of the data acquisition process, processing methods, and measures taken to ensure data quality.*

##### Response:

We have added some more details about the data sources and the process related to getting access and ensuring data quality, on page 4, line 79:

“We used linked population-wide administrative register data, covering information from several national registries. The registers were linked by Statistics Norway and then deidentified before researchers were allowed access. The acquisition of the data was part of the data structure at the Center for Fertility at the Norwegian Institute of Public Health, where data sets from the different registers were linked and stored at a secure server hosted by the Services for sensitive data (TSD), University of Oslo.

The information included in the database was demographic information and family status (national population register), labor market history (employer and employee register), educational attainment (national educational database), and physician visits (Norway Control and Payment of Health Reimbursements database). The data were accessed by the researchers for research purposes on March 1st, 2020.”

- *Explanation of analytical methods: For the RTI index and other statistical methods employed, offer more explanations and theoretical rationale to help readers better understand the applicability and advantages of these methods.*

##### Response:

We have explained the RTI index in some more detail to help the reader understand why this concept is meaningful for this type of analysis, on page 6, line 113:

“To measure structural risk of automation at the occupation level, we use a routine task intensity (RTI) index suggested in Acemoglu and Autor (2011). The measure is a theoretically informed indicator of structural economic risk, classifying occupations based on the tasks performed in it. In particular, the index captures the extent to which an occupation is characterized by routine tasks in the cognitive, manual or interpersonal domain. This reflects an economic theory of automation predicting that routine tasks are more easily automated, an assumption that has received extensive empirical support in work employing the RTI index (Autor,2003). Measurement of the content of job tasks were retrieved from the O*NET database. This database consists of a highly detailed description of tasks performed in an occupation. From the large set of measurements in O*NET, Acemoglu and Autor (2011) suggests a subset of items which describe these dimensions. To summarise these items to a

one-dimensional RTI measure for each occupation, we used the method suggested by Lewandowski et al. (2017) (see Supplementary material for details). Last, to ease interpretation, we standardized the RTI index distribution in the sample to be mean zero with a standard deviation of 1 (RTI z-score).”

- *Robustness checks: Although supplementary materials mention conducting robustness checks using different indicators, briefly mentioning these results in the main text would enhance the reliability of the conclusions.*

##### Response:

Robustness results are already noted both in the Methods section and in the results section:

Methods section: After presenting the RTI in the Methods section, since robustness concerns typically would be related to our measure of routine intensity, with the following text, on page 7, line 140:

“The main analysis in this paper is based on the 2003 version of the RTI – the first year of our study period. However, in the Supplementary material we also present all main results using both the FOI

and the RTI based on 2019 O*Net-data to assess robustness of our conclusions against changes in task content over time. S1 Table shows that all these measures of structural change are highly correlated, and our main conclusions are therefore independent of the selected measure.”

Results section - gender specific trends:

In the results section in relation to gender-specific trends we have the following, on page 11, line 223:

“In the Supplementary material we also present the average risk score using the Frey-Osbourne index (S2 Fig), RTI 2019 z-score (S3 Fig), and not z-standardized RTI scores (S4 Fig). It should be noted that when presenting time trends with the FOI or raw RTI-score, the time trend is downward sloping, indicating that the overall risk of automation is falling during the study period. However, the decrease is smaller for men, causing an increasing gender gap.”

Results section - in relation to risk factors:

On page 11, line 235:

“See the Supplementary material for models using the Frey-Osbourne index (S5 Fig) and RTI 2019 z- score (S6 Fig).”

4. *Results*
   - *Clarity in presentation of results: When presenting study results, use figures or tables to visually display the disparities between gender and socioeconomic status more intuitively.*

##### Response:

Based on this comment, and a more concrete comment made by another referee, we have thoroughly revised all legends and captions to ensure figures are consistent, self-explanatory and intuitive. We think this has been a substantial improvement.

This comment did not provide specific guidance on which aspects of our tables and figures needed revising, but our assessment was that legends and captions needed substantial adjustments.

5. *Discussion and Conclusion*
   - *Comprehensive discussion: In the discussion section, consider a more comprehensive examination of other potential explanatory factors, such as cultural and policy environments, to eliminate other potential confounding factors.*

##### Response:

This paper presents a description of how an occupation level measure of automation risk is associated with individual-level measures of socioeconomic status. This gives an overview of whether automation risks would plausible exacerbate existing inequalities. This is not a causal study, as clearly indicated in the title. There are hence no confounding factors in the standard sense given this design: if we find that people with low education and income are more likely to work in high RTI occupations this is so even if the individuals involved should turn out to have other protective factors not available to us in our data.

We have clarified this in the Strength and weaknesses section by noting that our results only assess how occupational RTI is correlated with the specific socioeconomic indicators used in our study and that other protective factors or risk factors may show different associations, on page 16, line 351 (addition highlighted):

”Second, the scope of the study is limited to what can be quantitatively measured in administrative registers and may not capture other risk factors or protective factors that could show different associations to occupational RTI scores, for example important experiential aspects related to

technological development that could be measured with qualitative methods.”

- *Clarity of conclusions: The conclusion should explicitly state the main findings, contributions, and implications for policymakers and researchers.*

##### Response:

We think the conclusion clearly presents our main findings. The comment gives no specific examples as to what issues the referee has with our conclusions and discussion or in what way they are too vague or could be improved. But, to become even more direct regarding policy implications, we have added the following, on page 16, line 359 (additions highlighted):

“...Given the increasing disparities identified in this study, policy makers should monitor the development in job market opportunities for various subgroups of the working-age population, and evaluate programs aimed at supporting individuals that are particularly negatively affected. For example, given the heightened risk faced by individuals with lower levels of education, policymakers should consider implementing targeted upskilling programs that equip these workers—especially younger individuals—with skills that are in high demand and less likely to be at risk of automation. Last, provided the recent rapid developments in generative AI, future research should also explore how the risk distribution is evolving over time across age-groups, industries and social risk factors.”

- *Future research directions: It would be beneficial to propose future research directions, such as further exploring differences across different age groups or industries, or studying the impact of technological changes on various social welfare indicators.*

##### Response:

We agree with this comment, and have added the following, on page 17, line 366:

“Last, provided the recent rapid developments in generative AI, future research should also explore how the risk distribution is evolving over time across age-groups, industries and social risk factors.”

##### Reviewer #4:

1. *Introduction: Ensure the introduction highlights the research gap more explicitly. This will better establish the context for the study and its relevance to the field. Furthermore, summarizing the hypothesis or key research questions can sharpen the focus.*

##### Response:

We agree that this aspect could be improved, and have taken following actions in response to your comment:

Research gap: we have added a clearer statement of our contribution in relation to the literature, on page 3, line 61:

“While previous studies primarily have focused on the negative impacts of automation on employment or inequality, less is known regarding the *distribution* of this risk across the population and to what extent we should expect it to dampen or amplify pre-existing socioeconomic differences. In particular, evidence on the development over time within subgroups and its association with other known risk factors of social marginalization, is scarce.”

Summarizing hypotheses: we added the following to the second paragraph of the introduction, on page 4, line 66:

“We hypothesize that men with lower education, who are more likely to be in routine-based occupations requiring fewer social skills, are at an increasingly higher risk of automation. Such a trend could exacerbate existing social inequalities [20].”

Key research question: we have rewritten research questions for increased precision, to the following, on page 4, line 69:

“The study aimed to investigate how a widely used indicator of occupational automation risk is associated and aligned at the individual level with measures of socioeconomic status in a register based study of the full population of Norwegian 45-year-olds, and how this differs for men and women. Our specific objectives were to (i) examine and explain the development in sex-specific risk across birth cohorts, and (ii) to estimate the association with other risk factors of social marginalization, including educational attainment, income, family background and health measures.”

*Methodology Clarification:*

1. *Clearly specify the reasoning behind using the RTI index over other measures of automation risk like the Frey-Osborne Index, even though both are discussed. Include a brief comparison in the methodology section, not just in the results section, to improve clarity for readers.*

##### Response:

In response to this comment we specified the reasoning behind our choice with the following, on page 6, line 113:

“To measure structural risk of automation at the occupation level, we use the routine task intensity (RTI) index suggested in Acemoglu and Autor (2011)[21]. This approach to measuring routine

intensity has been applied extensively in the labor economics literature on the impacts of skill based technological change, starting with the seminal contribution of Autor et al. (2003)[22].”

Then, later in the methodology section, we discuss briefly alternatives, on page 6, line 131:

“The RTI is one of two commonly used measures used to assess the risk of automation, with the other being the Frey-Osborne index (FOI) [22, 24]. The main difference between these measures is that the RTI is task-based – it assesses to what extent a job consists of tasks that could be automated – while the FOI is based on expert assessment of occupations. The measures are correlated, but RTI has a stronger theoretical foundation, allows for occupational scores to change with their task content [25], has done comparatively better in predictive tests [26], and has been used in an extensive literature on skill biased technological change [2, 22, 27-29] (see [30] and [31] for a description and discussion of the difference between these measures, and other alternative measures used in the literature).”

Our opinion is that this level of discussion is sufficient, and that more would decrease the readability of the paper. If the referee strongly disagrees, we are certainly willing to reconsider.

2. *Add more detail about how data access and linkage were done (e.g., anonymization procedures and encryption).*

##### Response:

We have added the following to better explain these aspects on page 4, line 79:

“We used linked population-wide administrative register data, covering information from several national registries. The registers were linked by Statistics Norway and then deidentified before researchers were allowed access. The acquisition of the data was part of the data structure at the Center for Fertility at the Norwegian Institute of Public Health, where data sets from the different registers were linked and stored at a secure server hosted by the Services for sensitive data (TSD), University of Oslo.

The information included in the database was demographic information and family status (national population register), labor market history (employer and employee register), educational attainment (national educational database), and physician visits (Norway Control and Payment of Health Reimbursements database). The data were accessed by the researchers for research purposes on March 1st, 2020.”

*Data Presentation:*

1. *Ensure consistency in reporting the variables. The terms used for categories (e.g., education, income) should remain consistent throughout.*

##### Response:

Thank you for bringing this to our attention. In the revised manuscript we have revised labeling of education levels and income both in the manuscript and supplementary information to ensure consistency.

(Note, as mentioned on page 8, line 171, income variables are not reported in the table since these are divided in quintiles - it does not make sense to report its distribution, since it has been created by us.)

2. *Consider providing more detailed tables or visual aids in the results section for critical variables to enhance clarity.*

##### Response:

We have thoroughly revised legends, captions and axis labels to provide a much better guide to the figures - this was a substantial improvement.

*Discussion Section:*

1. *Address the limitations more extensively, particularly focusing on the external validity of the findings outside of Norway.*

##### Response:

We have expanded the following to the limitations, on page 16, line 343:

“At the same time, our study has several weaknesses. First, the study has limited generalizability due to its focus on Norway, a country with unique characteristics related to gender roles, institutional environments, and industry structure. For example, Norway has an extensive welfare system that compensates 62.4 percent of the income in the case of job loss. Hence, the external validity of the results is limited, and likely most relevant for Northern European countries with similar institutions and social norms.”

2. *Expand on the implications of automation beyond just Norway to make the discussion more globally relevant. Including comparisons to other European countries or worldwide trends would enhance the broader significance of the findings.*

##### Response:

We have added the following, on page 16, line 348:

“At the same time, similar gender-based occupational patterns are likely to exist in diverse geographic contexts. Consequently, the widening gender gap in automation risk and its association with social risk factors are likely present in countries also far from Norway.“

*Figures and Tables:*

1. *Ensure all figures and tables are fully labelled and include clear legends. Some figures (like those comparing gender over time) could benefit from a more detailed description in the figure caption to ensure they are standalone and clear to readers.*

##### Response:

Thank you for this important comment. We have thoroughly revised all legends, captions, and axis labels to ensure figures are consistent, self-explanatory and intuitive. We have also revised some figures to improve clarity, e.g. axis labels of Fig 1.

2. *Ethical Considerations: Although the ethical aspects are addressed, adding a brief mention of any steps taken to minimize bias or stigmatization would further enhance transparency in research ethics.*

##### Response:

We agree that this aspect could be improved, and have added the following, on page 10, line 206: “To achieve this, we used neutral language and avoided portraying vulnerable groups in ways that

might be perceived as burdensome. Additionally, we refrained from reporting on groups small enough

to risk identifying individuals.“

*Conclusions: Strengthen the conclusions by linking the findings more explicitly to potential policy responses or interventions. Mention any ongoing or future research that might address gaps left by this study. This would also help connect the study’s relevance to broader public and governmental policies.*

##### Response:

We have added the following to the conclusions, on page 16, line 359 (additions highlighted):

“...Given the increasing disparities identified in this study, policy makers should monitor the development in job market opportunities for various subgroups of the working-age population, and evaluate programs aimed at supporting individuals that are particularly negatively affected. For example, given the heightened risk faced by individuals with lower levels of education, policymakers should consider implementing targeted upskilling programs that equip these workers—especially younger individuals—with skills that are in high demand and less likely to be at risk of automation. Last, provided the recent rapid developments in generative AI, future research should also explore how the risk distribution is evolving over time across age-groups, industries and social risk factors.”
