# Supplementary material for: The distribution of technology induced job loss: Evidence from a population-wide study in Norway
Source: PLoS One. 2025 Apr 15;20(4):e0321072. doi: 10.1371/journal.pone.0321072 (PMC11999129; doi:10.1371/journal.pone.0321072)
Supplement: Attachment — Submitted filename: Response_letter_auresp_3.docx [file pone.0321072.s019.docx]

**Response letter**

We want to express our sincere gratitude to the two referees for their time and effort in commenting on our paper. This process, and these new comments, have led to a substantial improvement in both the clarity of writing and nuance in the presentation of our results.

Note. All line numbers refer to the tracked-changes pdf-version of the manuscript.

**Response to Reviewer #5**

**Comment:**

*Abstract line 1-28: The abstract seems unclear. Are you focusing on automation or gender differences? Please emphasize the most critical aspect of your manuscript.*

**Response:**

Thank you for an important comment. You share concerns regarding the abstract with the other reviewer, where the other reviewer wants a clearer structure in the abstract (introduction, methods, results, and conclusion).

Based on both your comments, we have rewritten the abstract as follows (line 20-38):

“Globalization and automation are leading to structural changes in labor markets, resulting in the polarization of employment opportunities. These shifts are raising concerns about growing earnings inequality and gender disparities, particularly in occupations characterized by routine cognitive and physical tasks. This study utilizes comprehensive individual-level data from Norway to analyze gender differences in the routine intensity of occupations. The findings reveal significant and growing gender disparities. These disparities are most pronounced among individuals with low socioeconomic status. The analysis further identifies increasing gender differences in educational attainment as the primary contributor to the growing gender differences. Our results highlight the role of educational inequality in driving labor market disparities, emphasizing the need for targeted policy interventions to address these gendered dynamics, particularly among lower socioeconomic groups.”

**Comment:**

*Line 32: The phrase "increasing return to education" does not flow well. Consider breaking it into two sentences or simplifying it.*

**Response:**

Rewritten accordingly (changes highlighted; line 40):

“In recent decades, skill-biased technological change has enabled firms to automate and offshore routine-intensive tasks. This process has resulted in the erosion of low-skilled and mid-level jobs, while simultaneously increasing returns to education [1-4].”

**Comment:**

*Line 33: What is OECD? Please provide the full form before introducing the abbreviation.*

**Response:**

Thank you for making us aware about this shortcoming.

Revised to (page 3, line 43):

“These trends are expected to continue. According to a study from the Organisation for Economic Co-operation and Development (OECD) covering 32 member countries, 14% of jobs were classified as highly automatable.”

**Comment:**

*Line 33: By what year are 14% of jobs expected to be at risk?*

**Response:**

This is an interesting question, but outside the scope of our present paper. The study, and this report, focuses on the extent to which jobs are automatable. The extent to which this potential for automation will be realized and how fast this will occur is complex. There are several reasons, including the role of legal frameworks needing adjustments, labor unions and other structural barriers that make the speed of such processes highly uncertain, as well as the cost and economics of the switch. The implementation of driverless cars is a good example of the complexities involved beyond the technical engineering problem.

**Comment:**

*Line 34: How will automation not cause changes in long-term unemployment? Please elaborate on this in the next sentence.*

**Response:**

We agree, this is important to include.

We have revised as follows (changes highlighted; line 46):

“The disappearance of routine-intensive occupations may not necessarily result in an increase in long-term unemployment, as it is also expected to generate new opportunities and product markets that could drive labor demand [6]. From a classical economics perspective, labor saving automation frees up labor for new tasks, though the reconfiguration of the economy may involve a slow and painful shift in the labor market structure [7, 8].”

**Comment:**

*Line 40: What are the pre-existing inequalities? Consider adding examples such as gender disparities or differences between individuals with low and high education levels for clarity.*

**Response:**

We agree, and have revised to the following (line 55):

“This raises the question of whether a continuation of these trends will serve to amplify or

dampen pre-existing inequalities [10, 11], such as differences between individuals with low and high education levels.”

**Comment:**

*Line 41: Rephrase this sentence in a more scientifically acceptable manner.*

**Response:**

We have revised to (line 58):

“Several studies have identified men in occupations requiring less formal education as a group that has experienced stagnation or declines in both wage levels and labor market participation [12, 13].”

**Comment:**

*Line 52: Does the negative impact affect low-skilled or high-skilled labor? Please clarify.*

**Response:**

Unfortunately, this is not entirely clear from the referred to study. It studies four occupational categories: machine operator, assembler/trade worker, office worker, and engineer/scientist, and finds significant and substantial results for assemblers.

But, we have elaborated to make it more clear what the study finds (page 4, line 70):

“Recent evidence from Norway's manufacturing sector, which leverages import data on industrial robots, suggests that occupational groups particularly vulnerable to automation experience a decline in employment share and wages but also show an increase in unionization. This suggests that labor unions may play a role in mitigating some of the negative impacts of automation [19].”

**Comment:**

*Line 82: Why did you choose individuals born between 1958 and 1973 and those who turned 45? Why were individuals under 45 not included?*

**Response:**

Thank you for highlighting this issue. We agree that it requires clarification. We wanted to study development over time, and complete employment records with occupational codes were available to use for the period 2003 to 2018. We then selected age 45 as a “reference point” because, by this stage, individuals have typically completed their education, found a partner, started families, and established their careers. Put differently, at this age, labor market and family outcomes are generally in a relatively steady state.

Including younger individuals could risk a misleading pattern due to early-career trajectories of highly educated individuals with low earnings. Also, many are working in routine-jobs while studying. Moreover, family outcomes are not yet well-established, and these also have a strong socioeconomic gradient where highly educated individuals are slower to have children and settle down.

We have rewritten to the following (line 103):

“Our study period covers 2003-2018, as this was the period where we had population wide records of occupational codes. Provided the considerable differences in early-career labor market trajectories and the timing of family establishment across education levels, we included individuals at age 45 in each year. This approach, choosing mid-career, minimizes the risk of misleading associations arising from such variations in timing, driven by education. Hence, our sample consisted of the full population of individuals born 1958-1973 who were employed in the year they turned 45 and living in Norway (N = 900,559).”

**Comment:**

*Line 182: Include a statistical section in the manuscript with details on how the data was curated, organized, and analyzed. Specify the tools used, describe assumption testing conducted, and explain how the results can be interpreted from these tools (e.g., RTI and other risk tools). Mention the assumption testing conducted for regression analysis.*

**Response:**

Based on your comment we have now fully rewritten the previous section “Empirical strategy and statistical analysis”, to the following, which we think matches much better what you suggest, reproduced here for your convenience (line 182):

### **“Statistical Methods**

All analyses were conducted using R, version 4.1.1.

#### **Data Curation and Organization**

Our sample included all individuals born between 1958 and 1973 who were employed in the year they turned 45 and resided in Norway. The database contains all registered employer-employee relationships in Norway each year, hence covering all employed wage earners. Occupational RTI scores were assigned to individuals based on their main occupation in their employment records at age 45.

#### **Statistical Models**

Our aim was to examine how the risk of automation at the occupational level was associated with gender and various measures of socioeconomic status. This was achieved by estimating RTI z-score in different bivariate and multivariate regression models, stratified by gender. Hence, with this approach we both estimate RTI z-scores, and assess to what extent RTI z-scores can be explained by our explanatory variables.

First, to assess time trends, we therefore estimated the year- and gender-specific average RTI z-scores using regression models. Year and gender were included as binary indicators, hence the approach is equivalent to the conditional means. Since we used binary indicators, the method is semi-parametric, not resting on strict assumptions related to functional form.

Second, we examined how the structural risk of automation (RTI z-score) was associated with our measures of socioeconomic status and health (see Data and analytical sample). These models also included birth cohort dummies to adjust for potential time trends.

Third, we performed an attribution analysis where we estimated how much of the increase in automation risk gender gap could be explained by the various covariates included in the regression models. This analysis was carried out in two steps. First, the gender-specific change in prevalence over the period 2003 to 2018 was calculated. Then, to estimate the contribution, we multiplied these changes by the regression coefficients from the multivariate model to estimate the relative impact of each risk factor to the evolving gender-specific risk of automation.

Finally, to provide a detailed description of the variation in occupational risk scores in the most current population of 45-year-olds in our data - birth cohort 1973 - we estimated the RTI z-score for each observed combination of covariate values, separately for each gender. The results were plotted, ordered from low to high, in a bubble scatter plot where the bubble size reflects the size of the covariate group. To examine how these patterns have evolved over time, we also reproduced the plot for the first birth cohort in our data (born in 1958).”

**Comment:**

*Line 269: The discussion section is too brief. Provide a more detailed description of the risk of automation, with references to automation risks in both developing and developed countries. Additionally, discuss which sectors are more or less likely to be affected.*

**Response:**

Thank you for this comment.

Based on your suggestion we rewrote the discussion (from the second paragraph an onwards), both addressing developed vs developing countries and sectorial differences. We think this discussion flows much better. Thank you.

The following was written (line 349-380):

“Our study of Norway provides insights into automation risks within advanced economies, characterized by high educational attainment, strong norms of gender equality, and a well-developed technical infrastructure. A recent study covering 47 countries identified technology adoption and workforce skill levels as key factors driving cross-country variation in routine task intensity [32]. Therefore, developing countries, where larger shares of the population lack formal education, training, or work experience with information and communication technologies, were found to be more exposed to automation risks.

Gender disparities in automation risk have been examined across various regions, with mixed findings regarding which gender is more vulnerable [31, 34-36]. In Norway, we found that the growing gender gap in automation risk is largely driven by rising educational attainment among women. Notably, Statistics Norway reported in 2019 that this gap in education continues to widen, and that in many municipalities the share of highly educated women is more than twice that of men [37]. This trend reflects the substantial increase in women’s education across the Nordic countries in recent decades. However, the trend of increasing gender differences has, to the authors’ knowledge, not yet been shown in population-wide data covering a long time period. As this trend is likely to persist, policymakers must understand its implications and design strategies to mitigate potential negative impacts.

Automation risk varies significantly across sectors, depending on the extent to which occupations consist of more routine tasks, or require interpersonal and emotional skills. The recent Future of Jobs Report by the World Economic Forum, finds that jobs within the care economy and technology sector are growing, while clerical secretarial workers are expected to see the largest declines. In short, industries requiring a combination of emotional intelligence, complex problem-solving, and adaptability will remain challenging to automate in the foreseeable future. However, even within sectors assumed to grow over the coming decades, certain tasks (e.g., diagnostic imaging in healthcare or administrative work in education) are increasingly automated. In summary, the structure of the labor markets, educational trajectories chosen by different genders, cultural norms, technological readiness, industrial composition, will together determine how countries and genders, and other subgroups of the population, are differentially affected by technological developments over the coming decades.”

**Response to Reviewer #6**

**Comment:**

*1. Please try and make the abstract structure. i.e., in sections Abstract, Materials and Methods, Results and Conclusion*

*If unstructured. Please quantitatively mention the “significant” findings of the result.*

**Response:**

Thank you for an important comment.

We have rewritten the abstract as follows (with your suggested structure in mind):

“Globalization and automation are leading to structural changes in labor markets, resulting in the polarization of employment opportunities. These shifts are raising concerns about growing earnings inequality and gender disparities, particularly in occupations characterized by routine cognitive and physical tasks. This study utilizes comprehensive individual-level data from Norway to analyze gender differences in the routine intensity of occupations. The findings reveal significant and growing gender disparities. These disparities are most pronounced among individuals with low socioeconomic status. The analysis further identifies increasing gender differences in educational attainment as the primary contributor to these trends. Our results highlight the role of educational inequality in driving labor market disparities, emphasizing the need for targeted policy interventions to address these gendered dynamics, particularly among lower socioeconomic groups.”

**Comment:**

*2. Regarding "low-education", Consider reframing*

**Response:**

Thank you for this comment. In the introduction and the conclusion, we reframed to the following (change highlighted):

In the Introduction (line 51):

“In fact, a recent OECD policy brief found that countries with higher investments in robotics experienced higher employment growth [9], but also heightened job instability among workers in roles requiring less formal education.”

And in the conclusion (line 406):

“For example, given the heightened risk faced by individuals in jobs that require less formal education, policymakers should consider implementing targeted upskilling programs that equip these workers—especially younger individuals—with skills that are in high demand and less likely to be at risk of automation.”

For figures, and elsewhere in the manuscript, we use low education (as defined in the methods section based on the ISCED classification), as we think “low education” remains the most succinct term, given our dichotomization. Moreover, this terminology is clearly defined in the methods section, ensuring that readers can fully understand its scope and meaning within our study.

**Comment:**

*3. Please mention the sampling technique and then proceed to describe the details of the sample*

**Response:**

As we use administrative register data, we have data on our full population of interest. In fact, we observe all registered employees in the Norwegian economy, and then reduce our data set to the subset of the working population that we believe to be informative for the analyses at hand (i.e., old enough to have established careers, long term jobs and families). We now clarify this as follows:

In the data and analytical sample section (line 103):

“Our study period covers 2003-2018, as this was the period where we had population wide records of occupational codes. Provided the considerable differences in early-career labor market trajectories and the timing of family establishment across education levels, we included individuals at age 45 in each year. This approach, choosing mid-career, minimizes the risk of misleading associations arising from such variations in timing, driven by education. Hence, our sample consisted of the full population of individuals born 1958-1973 who were employed in the year they turned 45 and living in Norway (N = 900,559).”

**Comment:**

*4. Please clarify marital status a bit more clearly, or add the fallacy in the limitation: Separated couples, problem families may not always report to the register, and the register may not reflect the change in social contract*

**Response:**

We added the following to explanation of this variable (line 161):

“Hence, separated couples, or couples that for other reasons did report to the register, were not captured by our measure.”

**Comment**:

*5.Regarding the final sentence in the Ethical considerations paragraph: Additionally, we*

*refrained from reporting on groups small enough to risk identifying individuals*

*“Small enough” is a vague term. Please mention what was the cutoff for considering a group “Small enough”*

**Response:**

We agree. This is too vague.

Changed to (line 233):

“Additionally, the study does not report on group sizes that could potentially disclose information about individual statistical units, in accordance with the recommendations outlined in the Handbook on Statistical Disclosure Control [32].”

**Comment:**

*6. Large language models may be abbreviated as LLMs as the usage is more than once in the manuscript*

**Response:**

We agree. Revised accordingly.

**Comment:**

*7. Consider reframing sentences corresponding to 328 to 331.*

**Response:**

We reframed as follows (changes highlighted; line 406):

“For example, given the heightened risk faced by individuals in jobs that require less formal education, policymakers should consider implementing targeted upskilling programs that equip these workers—especially younger individuals—with skills that are in high demand and less likely to be at risk of automation.”
